# Assessment and Implication of Prognostic Imbalance in Randomized Controlled Trials with a Binary Outcome – A Simulation Study

**DOI:** 10.1371/journal.pone.0036677

**Published:** 2012-05-22

**Authors:** Rong Chu, Stephen D. Walter, Gordon Guyatt, P. J. Devereaux, Michael Walsh, Kristian Thorlund, Lehana Thabane

**Affiliations:** 1 Department of Clinical Epidemiology and Biostatistics, Faculty of Health Sciences, McMaster University, Hamilton, Ontario, Canada; 2 Population Health Research Institute, Hamilton Health Sciences, Hamilton, Ontario, Canada; 3 Biostatistics Unit, Father Sean O'Sullivan Research Centre, St Joseph's Healthcare Hamilton, Hamilton, Ontario, Canada; 4 The Centre for Evaluation of Medicines, St Joseph's Healthcare Hamilton, Hamilton, Ontario, Canada; University of Florida, United States of America

## Abstract

**Background:**

Chance imbalance in baseline prognosis of a randomized controlled trial can lead to over or underestimation of treatment effects, particularly in trials with small sample sizes. Our study aimed to (1) evaluate the probability of imbalance in a binary prognostic factor (PF) between two treatment arms, (2) investigate the impact of prognostic imbalance on the estimation of a treatment effect, and (3) examine the effect of sample size (n) in relation to the first two objectives.

**Methods:**

We simulated data from parallel-group trials evaluating a binary outcome by varying the risk of the outcome, effect of the treatment, power and prevalence of the PF, and n. Logistic regression models with and without adjustment for the PF were compared in terms of bias, standard error, coverage of confidence interval and statistical power.

**Results:**

For a PF with a prevalence of 0.5, the probability of a difference in the frequency of the PF≥5% reaches 0.42 with 125/arm. Ignoring a strong PF (relative risk = 5) leads to underestimating the strength of a moderate treatment effect, and the underestimate is independent of n when n is >50/arm. Adjusting for such PF increases statistical power. If the PF is weak (RR = 2), adjustment makes little difference in statistical inference. Conditional on a 5% imbalance of a powerful PF, adjustment reduces the likelihood of large bias. If an absolute measure of imbalance ≥5% is deemed important, including 1000 patients/arm provides sufficient protection against such an imbalance. Two thousand patients/arm may provide an adequate control against large random deviations in treatment effect estimation in the presence of a powerful PF.

**Conclusions:**

The probability of prognostic imbalance in small trials can be substantial. Covariate adjustment improves estimation accuracy and statistical power, and hence should be performed when strong PFs are observed.

## Introduction

Because randomization attempts to balance the distribution of known and unknown prognostic factors (PFs) between treatment groups, authorities view it as critical for ensuring unbiased assessment of treatment effects [1]. Despite randomization, imbalance in PFs as a result of chance (chance imbalance) may still arise, and with small to moderate sample sizes such imbalance may be substantial [2], [3]. Ignoring chance imbalance in key PFs between treatment groups may result in a biased estimate of the treatment effect, particular when a large between-group difference occurs in a powerful PF [4]–[7].

Control for unbalanced PFs is often achieved via statistical techniques such as regression analysis, sometimes in conjunction with other design features such as stratified randomization. Adjusting for balanced or marginally unbalanced PFs of high predictive value increases statistical power and reduces sample size requirements [8]–[13]. While including balanced baseline covariates in linear models does not change the estimate of treatment effect, omitting balanced covariates in logistic regression models may lead to underestimation of subject-specific treatment effects [14]–[16]. Although guidelines for RCTs recommend conducting both unadjusted and adjusted analyses [17]–[19], only a minority of trials report adjusted analyses [13], [20]. Moreover, although recommendations also suggest specifying key PFs in the protocol based on prior judgement, there is often insufficient prior knowledge to ascertain all important PFs before a trial commences [13], [21].

Sample size of RCTs plays a critical role in balancing known and unknown PFs between treatment groups. Although many clinical trials with a binary outcome employ power calculations to determine an adequate sample size, underpowered studies are common [22]–[24]. Among 519 PubMed-indexed RCTs published in December 2000, the median total sample size per trial was 52 (10^th^–90^th^ percentile: 12–310) considering all designs and 80 (10^th^–90^th^ percentile: 25–369) considering only parallel-group trials [25]. A more recent systematic review of 215 two-arm parallel group RCTS of superiority with a single primary outcome published in six high impact factor general medical journals between January 1, 2005 and December 31, 2006 indicates a larger median total trial size of 425 (interquartile range: 158–1041) [26]. Sample size calculations often assume a balance of prognosis between the treatment groups regardless of sample size, yet the distribution of the possible unobserved PFs can be difficult to examine using empirical data mainly because they are unobserved.

The current simulation study was designed to address three objectives: (1) to evaluate the probability of imbalance in a binary PF between two treatment groups in simple RCTs with standard randomization (without stratification, blocking or minimization) evaluating a binary outcome; (2) to investigate the impact of prognostic imbalance on the estimation of treatment effect; and (3) to examine the effect of sample size on the probability and impact of prognostic imbalance in RCTs.

## Methods

### Simulation framework

We considered parallel group RCTs with a binary outcome in which equal numbers of patients were randomized to the treatment and control groups. For simplicity, we confined our attention to only one baseline PF without stratification. Five trial design parameters were considered: the frequency of the outcome event in the control group; the effect of treatment on the outcome; the strength of the association between the PF and the outcome; the prevalence of the PF; and the sample size.

We explored two simulation settings. For setting #1, we did not impose any level of imbalance, but simply generated a binary PF (C = 0, 1) independently from the treatment allocation (T = 0, 1) for each simulated trial. We refer to this as the “unconditional setting”. This setting allowed us to evaluate the cumulative probability of prognostic imbalance greater than or equal to some level, and address whether or not adjusting for a baseline PF that is subject to chance imbalance improves the accuracy, precision and efficiency of the estimation of treatment effects.

We refer to setting #2 as the “conditional setting” for which we imposed a particular level of imbalance in each simulated trial, specifically, 5% more patients in the control group having the PF than those in the treatment group. Although, over a large number of RCTs, the probability of repeated occurrence of imbalance approaches zero, the conditional setting allowed us to explore what would happen if there were a 5% imbalance in a particular trial. This provided a way to assess the magnitude of potential bias resulting from an imbalance if it was unobserved or omitted from the analysis. It also allowed us to study whether this potential bias could be controlled by increasing the sample size.

#### Setting #1: the unconditional setting

Each simulated dataset in the unconditional setting consisted of a binary indicator for treatment allocation (T = 0, 1), a binary baseline PF (C = 0, 1), and a binomial response variable (Y), indicating the number of patients who experience an outcome event (D = 0, 1) for each T-C categorization. We related the log odds of experiencing the outcome D = 1 conditional on the allocated treatment and baseline prognosis through the following model:

Simulation model:
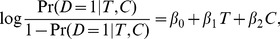
(1)where 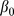 corresponds to the log odds of the outcome among patients without the PF in the control group, 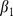 corresponds to the log odds ratio (OR) of having the outcome in the experimental treatment group relative to the control group conditional on baseline prognosis (i.e. the treatment effect), and 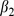 corresponds to the log OR of the outcome among patients having the PF versus not conditional on treatment status.

We assumed equal numbers of patients being randomized to the experimental group (T = 1) and control group (T = 0), i.e. n_1_ = n_0_ = n. We sampled C independently of T from the binomial distribution Bin(n_i_, λ), with prevalence λ being fixed at 14 values between 0.005 and 0.995, namely, 0.005, 0.01, 0.05, 0.1, 0.2, 0.3, 0.4, 0.5, 0.6, 0.7, 0.8, 0.9, 0.95, and 0.995. We simulated 8 scenarios (Table 1) by varying each of three parameters to reflect typical features of a cardiovascular prevention trial: (a) risk of outcome event in the control group (a low risk of 0.05; and a moderate risk of 0.10), (b) treatment effect size in the absence of the PF (a moderate effect: relative risk [RR] of 0.75; and a zero effect: RR of 1), and (c) effect of the PF on the outcome in the control group (a strong effect: RR of 5; and a moderate effect: RR of 2). If the covariates are strongly predictive of the outcome, i.e. strong PFs, mild or moderate imbalance can result in a biased effect estimate [3], [27]. The potential impact of dissimilarity in such strong PFs between groups can plausibly be greater when the risk of event in the control group is low, because results of hypothesis testing may be more sensitive to the change in the numbers of outcome events in treatment groups when the outcomes are rare. For each scenario, we investigated six sample sizes and the 14 λ values listed above. Considering a clinical trial aiming to detect a moderate treatment effect (i.e. RR = 0.75) and a moderate risk of the outcome in the control group (i.e. 0.10), a standard power calculation suggests a total of 4000 patients (2000 per group) is needed to yield type I and type II error rates of 5% and 20%, respectively. To assess the impact of sample size on prognostic imbalance, we also included ½, ¼ and 1/16 of this calculated sample size for each simulation scenario (corresponding to 1000, 500 and 125 patients per arm, respectively). We also considered two smaller sample sizes (25 and 50 patients per arm) because small trials occur frequently in medical publications [25]. We simulated 10,000 trials per prevalence per sample size per scenario.

**Table 1:** Simulation scenarios for the unconditional and conditional settings.

Scenario	Effect of treatment in RR* (B1)	Effect of PF in RR† (B2)	Incidence of outcome (B0)	Prevalence of PF (C)	Sample size/arm
1	0.75 (−0.315)	5 (2.197)	0.1 (−2.197)		
2	0.75 (−0.315)	2 (0.811)		0.005–0.995	(a) 25
3	1 (0)	5 (2.197)		(unconditional)	(b) 50
4	1 (0)	2 (0.811)			(c) 125
5	0.75 (−0.301)	5 (1.846)	0.05 (−2.944)	0.05–0.95	(d) 500
6	0.75 (−0.301)	2 (0.747)		(conditional)	(e) 1000
7	1 (0)	5 (1.846)			(f) 2000
8	1 (0)	2 (0.747)			

* *Relative risk of having an outcome event for people receiving the experimental treatment (vs. control treatment) without the prognostic factor.*

† *Relative risk of having an outcome for people with vs. without the PF in the control group.*

#### Setting #2: the conditional setting

We also simulated 10,000 replicates for each combination of the prevalence and sample size per scenario as per Table 1 in the conditional setting. For each trial, 5% more patients had the PF in the control group than the treatment group. We fixed the overall proportion of the PF at each of the 11 values: 0.05, 0.1, 0.2, 0.3, 0.4, 0.5, 0.6, 0.7, 0.8, 0.9, and 0.95, as the probability of observing a 5% imbalance is extremely low for λ<0.05 or >0.95. We conducted all simulations and analyses in ***R*** 2.12.1.

### Analysis

#### Distribution of imbalance

For each scenario, we retained the simulated proportion of patients with the PF per arm, with continuity correction by adding 0.5 to each T-C categorization to handle sparse cells [28]. We quantified imbalance using two different measures: the absolute difference (D_1_) and the standardized difference (D_2_), as follows:

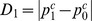
, and

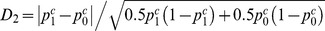
,

where p^c^_i_ = proportion of patients having the PF (C = 1) (with continuity correction) at baseline in the treatment (i = 1) or control (i = 0) group. We decided to use the absolute difference, D_1_, as the primary measure of imbalance, because it is more intuitive for clinicians. The standardized measure has been advocated for having better statistical properties and may be more appealing to the statistical audience [29], [30]. We assessed the probabilities of observing different levels of imbalance for each sample size: Pr (D_i_≥*d_1_*), where *d_1_* = 0.005, 0.01, 0.025, 0.05, 0.10, 0.15, 0.20.

#### Impact of prognostic imbalance on treatment effect estimation

We fit two logistic regression models to evaluate the effect of treatment with and without adjustment for the PF. The adjusted model was the same as the underlying simulation model (Eq. 1) and the unadjusted model took the form of Eq. 2.

Unadjusted Model:
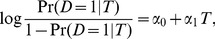
(2)where 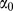 represents the log odds of having the outcome among patients in the control group (with or without the PF), and 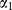 represents the log OR of the outcome in the experimental treatment group relative to the control group regardless of baseline prognosis.

For each simulated RCT, we recorded the estimated regression coefficients, their associated estimated standard errors (SEs), 95% confidence intervals (CIs, based on Ward test), and fitted probabilities of the outcome for each T-C (for the adjusted model) or C (for the unadjusted model) categorization. For each scenario, bias of the estimated regression coefficient (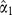 or 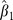) relative to the true log OR (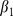), its empirical standard deviation (SD), and mean squared error (MSE) were recorded for each model. The empirical coverage of the 95% CI was computed as the proportion of CIs that contained the true effect; and power was calculated as the proportion of replications where the CI excluded the null.

## Results

### Distribution of imbalance

**Figure 1:**
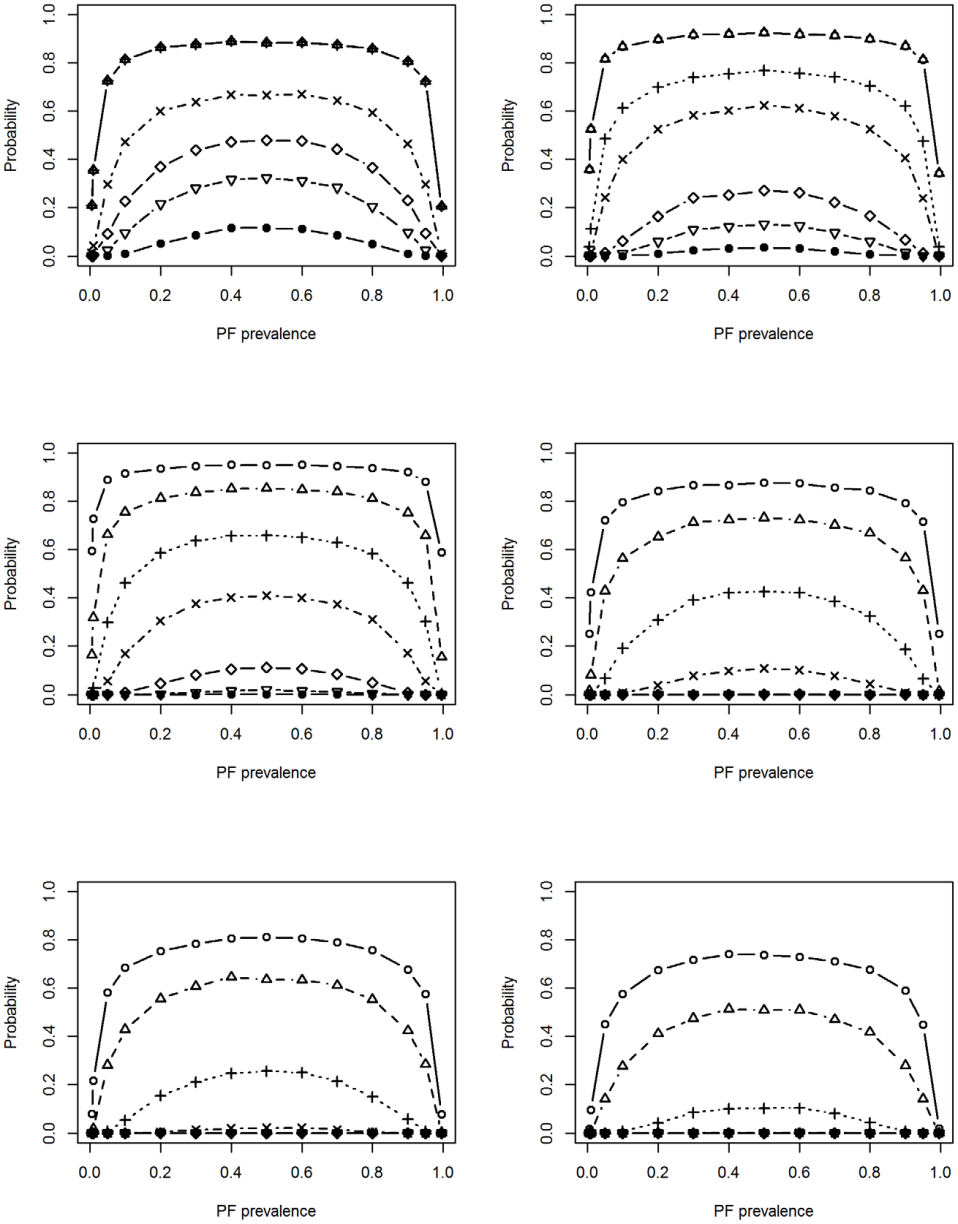
Probability of imbalance using absolute measure (D_1_) with different trial sizes. Lines correspond to Pr (D_1_≥d_1_), where d_1_ = 0.005 (hollow circle), 0.01 (triangle), 0.025 (cross), 0.05 (X), 0.10 (diamond), 0.15 (inverted triangle), and 0.20 (filled circle), from the top to the bottom, respectively. Top left: 25/arm, top right: 50/arm, middle left: 125/arm, middle right: 500/arm, bottom left: 1000/arm, bottom right: 2000/arm.

Figure 1 displays the cumulative probabilities of an imbalance using the absolute measure (D_1_) with 25, 50, 125, 500, 1000, and 2000 patients per arm. For a fixed sample size, the probability of imbalance varied with the prevalence of the PF (λ): imbalance was more likely to occur when λ is close to 0.5, but probability diminished as λ approached 0 or 1. The probability of imbalance increased markedly as sample size decreased regardless of λ. For a PF with prevalence of 0.5, the probability of an imbalance ≥5% was about 0.02 with 1000 patients per arm, 0.1 with 500 patients per arm, and 0.42, 0.62 and 0.67 with 125, 50 and 25 patients per arm, respectively. When the prevalence of PF was 0.05, Pr(D_1_≥0.05) increased from ≤0.0001, 0.0004, 0.059, 0.24 and 0.29 as sample size decreased from 1000 to 25.

Figure S1 displays the cumulative probability of imbalance using the standardized measure (D_2_). Because the absolute difference was scaled by the pooled SD to create the standardized measure, λ had little impact on the probability of D_2_, except for the extreme values. Common for both imbalance measures, the chance of imbalance decreased with increasing sample size. However, the relationship between the probability of imbalance and the prevalence of the PF differed using different measures.

### Impact of prognostic imbalance on treatment effect estimation

#### Setup #1: the unconditional setting

**Figure 2:**
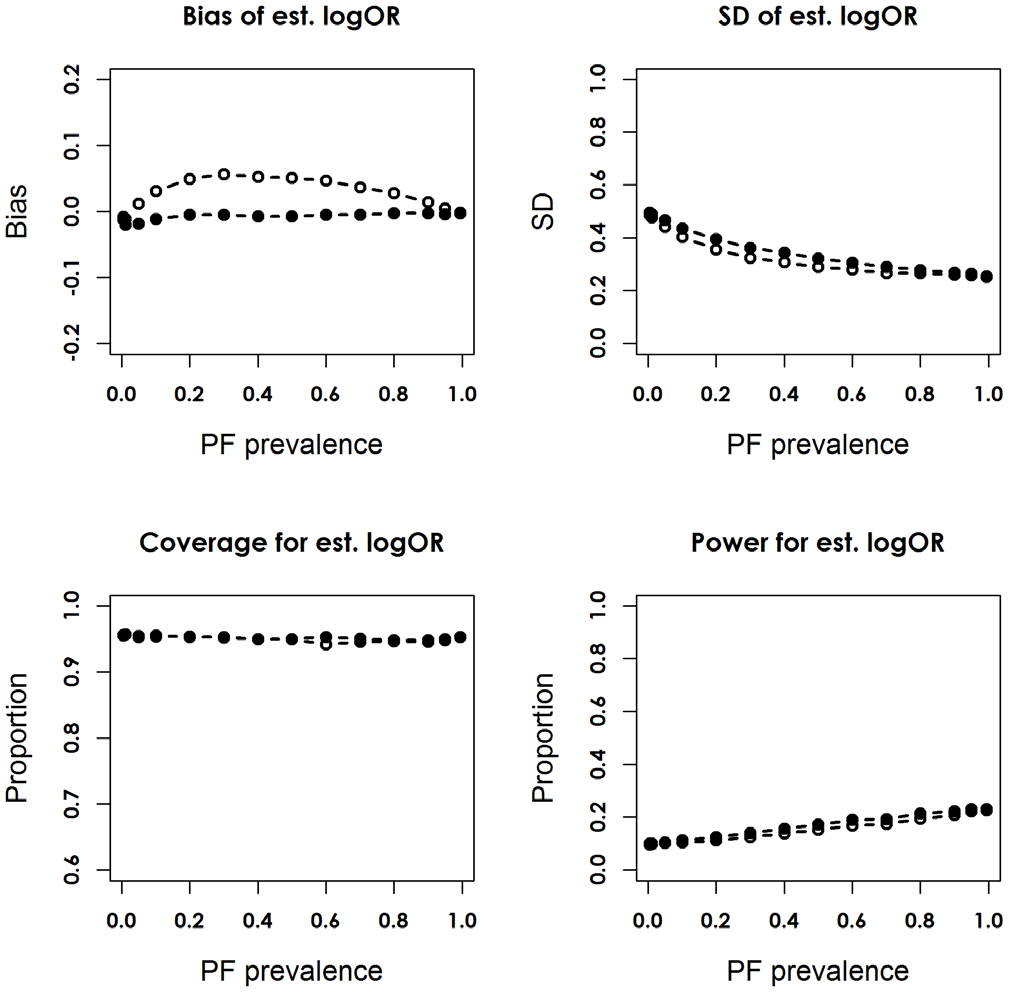
Bias, simulation standard deviation (SD), coverage proportion and statistical power for the unadjusted and adjusted logOR, in scenario 1, the unconditional setting, with 125 patients per arm. The unadjusted model is indicated by the dotted line with hollow circles, and the adjusted model is indicated by the solid line with filled circles.

**Figure 3:**
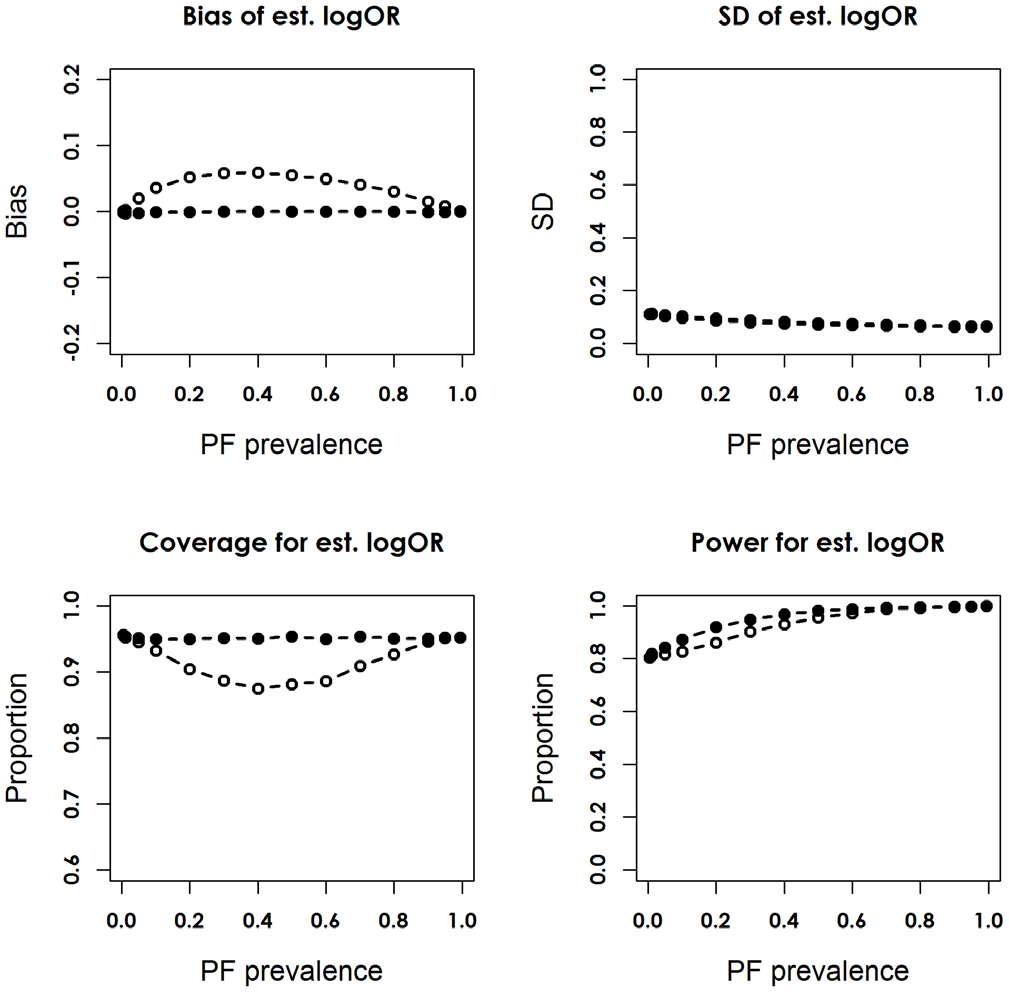
Bias, simulation standard deviation (SD), coverage proportion and statistical power for the unadjusted and adjusted logOR, in scenario 1, the unconditional setting, with 2000 patients per arm. The unadjusted model is indicated by the dotted line with hollow circles, and the adjusted model is indicated by the solid line with filled circles.

Scenario 1 corresponded to trials with a 10% risk of the outcome in the control group, a strong prognostic factor (RR = 5), and a moderate treatment effect (RR = 0.75, corresponding OR = 0.73) (Table 1). Figures 2 and 3 depict the bias and empirical SD of the point estimator of log OR, the coverage of the 95% CI, and the empirical statistical power for the adjusted and unadjusted models with 125 and 2000 patients per arm. When PF was omitted from the logistic regression, the estimated log OR was biased towards zero.

The magnitude of bias declined as λ approached 0 or 1, but varied little with sample size when each arm had 50 or more patients. The adjusted estimator 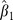 was unbiased conditional on baseline prognosis, and independent of λ and sample size, when there were over 50 patients per arm. With 25 patients per arm, estimated log ORs from both models tended to be biased towards zero for λ≤0.1; the adjusted estimates were slightly negatively biased for greater λ values. This was possibly due to the lack of outcome events to reliably estimate the treatment contrast (Figures S2 and S3).

Adjusting for the PF reduced precision of the point estimator, especially when the trial size was less than 500 per arm. The adjusted model was able to maintain the nominal coverage of the 95% CI for different trial sizes. In contrast, coverage of the unadjusted model was less than the nominal value for most λ values, when sample size exceeded 500 per arm; the decline was more drastic when λ was near 0.5. For a PF with prevalence of 0.5, the actual coverage of the unadjusted 95% CIs was 95%, 93.58%, 91.15%, and 88.12% with 25–125, 500, 1000 and 2000 patients per arm, respectively.

When λ decreased to 0.05, coverage of the unadjusted 95% CIs was roughly around the nominal value. Despite a slight loss of precision, the adjusted model had equal or greater statistical power across of the spectrum of the prevalence of PF. The gain in power was more marked when sample size was between 500 and 1000 per arm (Figures S4 and S5), probably due to the floor or ceiling effect associated with very small or large sample sizes, i.e. power from both models approached 0 or 100%, so the difference in power between models shrank accordingly.

For a PF with prevalence of 0.5, the loss of power of the unadjusted model relative to the adjusted model was 3.44%, 15.20%, 11.29%, 14.39%, 9.66%, and 2.61% with 25, 50, 125, 500, 1000 and 2000 patients per arm. The two models achieved similar power for a rare PF with λ<0.1. For both models, the precision of point estimator and empirical power increased with the number of outcome events resulted from increasing sample size and λ.

As relative risk of experiencing an outcome event for those with the PF versus those without in the control group reduced from 5 to 2 (scenario 2), bias associated with the unadjusted point estimator of log OR became negligible for all trial sizes (except for 25 per arm with λ≤0.2). The adjusted and unadjusted models were also similar in terms of precision, coverage of CI and statistical power (Figures S6, S7, S8, and S9).

When the treatment had no effect on the outcome of interest (scenarios 3 and 4), the adjusted and unadjusted models produced unbiased point estimate despite the predictive power of the PF. Adjusting for baseline PF was not necessary in this situation to remove bias, and in fact it led to a slight inflation of SD. Sample size had little impact on the comparative performance of the two models, and nominal coverage of CI was achieved for both models (Figure S10).

For scenarios 5–8, where there was 5% risk of the outcome in the control group, the results demonstrated patterns similar to those described above for the first four scenarios. Precision of the point estimates and statistical power were lower for both models in scenarios 5–8. The magnitude of bias of the unadjusted log OR estimator in scenario 5 was slightly less than those in scenario 1. Differences in statistical power between two models also decreased slightly with the risk of outcome event when a treatment difference truly existed.

**Figure 4:**
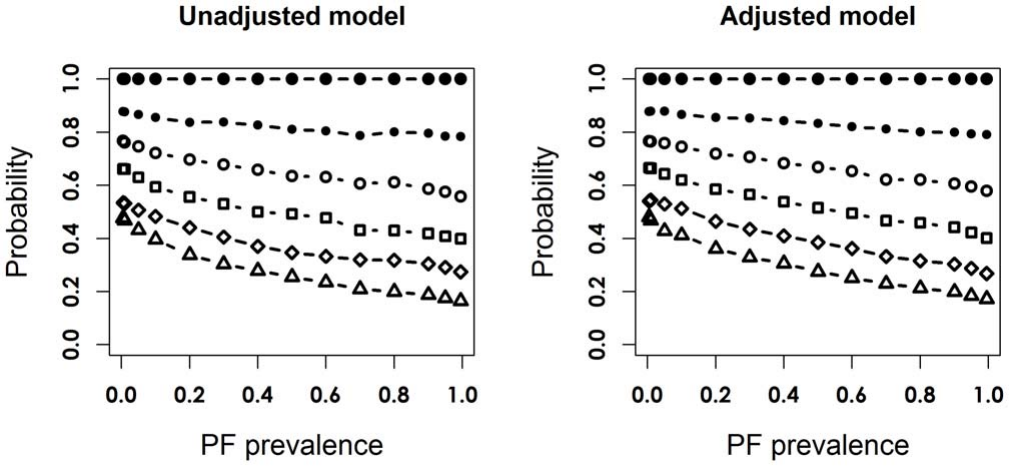
Probability of difference between the estimated and true ORR (deviation in either direction) in scenario 1, the unconditional setting, with 125 patients per arm. Within each graph, lines correspond to Pr (|D_ORR_|≥d_2_), where d_2_ = 0 (solid circle), 0.05 (bullet), 0.10 (little circle), 0.15 (square), 0.2 (diamond) and 0.25 (triangle), from top to bottom, respectively.

**Figure 5:**
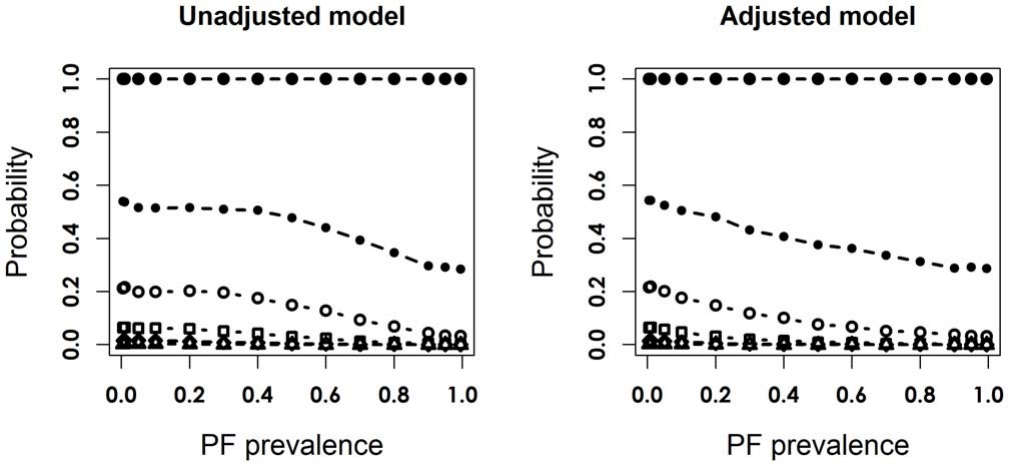
Probability of difference between the estimated and true ORR (deviation in either direction) in scenario 1, the unconditional setting, with 2000 patients per arm. Within each graph, lines correspond to Pr (|D_ORR_|≥d_2_), where d_2_ = 0 (solid circle), 0.05 (bullet), 0.10 (little circle), 0.15 (square), 0.2 (diamond) and 0.25 (triangle), from top to bottom, respectively.

Figure 4 and Figures S11 and S12 display distributions of the differences (*D_ORR_*) between the estimator of OR reduction (ORR, defined as 1 - OR) and the true ORR, i.e. 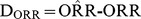, across the spectrum of λ, based on 10,000 trials in scenario 1, with 125 patients per arm. The vertical axis represents the proportion of trials associated with a difference greater than or equal to a certain value *d_2_*, where *d_2_* = 0, 0.05, 0.10, 0.15, 0.2 or 0.25. While Figure 4 corresponds to the probability of deviations in either direction, 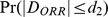, Figures S11 and S12 correspond specifically to underestimation, 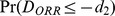 and overestimation, 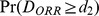, respectively. Figure 5 and Figures S13 and S14 present distributions of *D_ORR_* for the same scenario with 2000 per arm. Tables 2 and 3 present the proportions of difference at selected λ values across all sample sizes in scenario 1.

**Table 2:** Probability of difference between the estimated and true ORR≥0.05 in the unconditional setting scenario 1.

Difference from true ORR≥0.05		Unadjusted model					Adjusted model				
	Sample size	Prevalence of PF					Prevalence of PF				
		0.01	0.05	0.1	0.2	0.5	0.01	0.05	0.1	0.2	0.5
Over-	25	0.45	0.45	0.44	0.44	0.44	0.45	0.44	0.46	0.48	0.47
estimation	50	0.46	0.44	0.43	0.42	0.39	0.47	0.47	0.47	0.47	0.45
	125	0.44	0.42	0.39	0.35	0.33	0.45	0.45	0.44	0.43	0.42
	500	0.37	0.33	0.29	0.24	0.20	0.38	0.38	0.37	0.36	0.33
	1000	0.32	0.27	0.21	0.16	0.11	0.33	0.33	0.31	0.29	0.26
	2000	0.25	0.19	0.14	0.08	0.04	0.27	0.26	0.25	0.23	0.18
Under-	25	0.52	0.50	0.48	0.47	0.51	0.52	0.51	0.48	0.46	0.46
estimation	50	0.47	0.49	0.49	0.47	0.49	0.46	0.46	0.46	0.44	0.45
	125	0.43	0.45	0.46	0.48	0.48	0.43	0.43	0.43	0.43	0.41
	500	0.39	0.41	0.44	0.46	0.47	0.39	0.37	0.37	0.36	0.33
	1000	0.34	0.37	0.42	0.46	0.46	0.33	0.32	0.32	0.31	0.28
	2000	0.28	0.33	0.38	0.44	0.44	0.27	0.26	0.26	0.25	0.20
Overall	25	0.96	0.94	0.92	0.91	0.95	0.97	0.95	0.94	0.94	0.93
	50	0.93	0.92	0.92	0.89	0.88	0.93	0.93	0.93	0.91	0.90
	125	0.88	0.87	0.86	0.84	0.81	0.88	0.88	0.87	0.86	0.83
	500	0.76	0.75	0.73	0.70	0.66	0.76	0.75	0.74	0.72	0.67
	1000	0.66	0.64	0.63	0.62	0.57	0.66	0.65	0.63	0.60	0.54
	2000	0.54	0.52	0.51	0.52	0.48	0.54	0.52	0.51	0.48	0.38

*ORR: odds ratio reduction; PF: prognostic factor.*

**Table 3:** Probability of difference between the estimated and true ORR≥0.10 in the unconditional setting scenario 1.

Difference from true ORR≥0.10		Unadjusted model					Adjusted model				
	Sample size	Prevalence of PF					Prevalence of PF				
		0.01	0.05	0.1	0.2	0.5	0.01	0.05	0.1	0.2	0.5
Over-	25	0.37	0.37	0.38	0.40	0.38	0.39	0.41	0.43	0.44	0.44
estimation	50	0.40	0.39	0.39	0.38	0.33	0.42	0.43	0.43	0.43	0.39
	125	0.38	0.35	0.32	0.28	0.24	0.39	0.38	0.37	0.35	0.33
	500	0.26	0.22	0.17	0.12	0.08	0.27	0.25	0.24	0.22	0.18
	1000	0.17	0.13	0.09	0.05	0.02	0.18	0.18	0.16	0.14	0.09
	2000	0.09	0.05	0.03	0.01	0.00	0.10	0.09	0.07	0.07	0.03
Under-	25	0.51	0.49	0.47	0.43	0.42	0.52	0.49	0.46	0.43	0.42
estimation	50	0.41	0.43	0.44	0.45	0.42	0.41	0.43	0.42	0.40	0.40
	125	0.39	0.40	0.41	0.42	0.39	0.38	0.38	0.38	0.37	0.34
	500	0.29	0.30	0.31	0.32	0.30	0.28	0.27	0.26	0.24	0.20
	1000	0.21	0.23	0.25	0.26	0.24	0.20	0.20	0.19	0.17	0.13
	2000	0.13	0.15	0.17	0.19	0.15	0.12	0.11	0.10	0.08	0.05
Overall	25	0.88	0.86	0.85	0.83	0.80	0.91	0.90	0.89	0.88	0.86
	50	0.81	0.81	0.83	0.82	0.75	0.83	0.85	0.85	0.83	0.79
	125	0.76	0.75	0.72	0.70	0.63	0.76	0.76	0.75	0.72	0.67
	500	0.55	0.52	0.49	0.45	0.38	0.55	0.52	0.50	0.47	0.39
	1000	0.38	0.36	0.34	0.32	0.26	0.38	0.37	0.35	0.30	0.22
	2000	0.22	0.20	0.20	0.20	0.15	0.22	0.20	0.18	0.15	0.08

*ORR: odds ratio reduction; PF: prognostic factor.*

Overall, the proportion of random deviations decreased when the sample size, λ and the size of the deviation increased. When λ = 0.05 in scenario 1, the probabilities of *D_ORR_*≥0.05 (in either direction) from the true ORR was 0.87–0.88 and 0.52 with 125 and 2000 patients per arm, respectively, for both models (Table 2). In comparison, the probabilities of *D_ORR_*≥0.1 dropped to 0.75–0.76 (125/arm) and 0.20 (2000/arm) at the same prevalence (Table 3). When the treatment effect was zero, the corresponding probabilities of a given deviation were higher (Tables 4 and 5). For instance, probabilities of *D_ORR_*≥0.1 were 0.78–0.81 (125/arm) and 0.30–0.32 (2000/arm) when λ = 0.05 in scenario 3. The probabilities of *D_ORR_*≥0.1 remained above 0.8 with 50 or less patients per arm in all scenarios, when λ was between 0.01 and 0.5.

**Table 4:** Probability of difference between the estimated and true ORR≥0.05 in the unconditional setting scenario 3.

Difference from true ORR≥0.05		Unadjusted model					Adjusted model				
	Sample size	Prevalence of PF					Prevalence of PF				
		0.01	0.05	0.1	0.2	0.5	0.01	0.05	0.1	0.2	0.5
Over-	25	0.39	0.41	0.41	0.42	0.43	0.39	0.44	0.47	0.48	0.47
estimation	50	0.44	0.44	0.44	0.45	0.46	0.44	0.47	0.47	0.47	0.46
	125	0.45	0.46	0.46	0.45	0.42	0.46	0.45	0.45	0.45	0.44
	500	0.41	0.41	0.39	0.39	0.36	0.33	0.32	0.31	0.30	0.27
	1000	0.36	0.35	0.35	0.33	0.31	0.37	0.36	0.36	0.35	0.32
	2000	0.31	0.30	0.29	0.27	0.23	0.31	0.30	0.30	0.29	0.26
Under-	25	0.40	0.41	0.41	0.43	0.44	0.41	0.45	0.47	0.47	0.48
estimation	50	0.43	0.43	0.45	0.45	0.45	0.43	0.46	0.47	0.47	0.46
	125	0.46	0.46	0.46	0.45	0.42	0.46	0.45	0.45	0.44	0.44
	500	0.40	0.40	0.39	0.38	0.35	0.41	0.40	0.40	0.39	0.37
	1000	0.37	0.36	0.35	0.34	0.31	0.37	0.37	0.35	0.35	0.33
	2000	0.32	0.31	0.30	0.28	0.24	0.32	0.32	0.31	0.29	0.26
Overall	25	0.79	0.81	0.82	0.85	0.87	0.80	0.89	0.93	0.95	0.95
	50	0.86	0.88	0.89	0.90	0.91	0.87	0.93	0.94	0.94	0.92
	125	0.92	0.93	0.92	0.91	0.83	0.92	0.90	0.90	0.89	0.88
	500	0.81	0.81	0.78	0.77	0.71	0.73	0.72	0.71	0.69	0.64
	1000	0.73	0.71	0.70	0.67	0.61	0.73	0.73	0.72	0.70	0.65
	2000	0.63	0.60	0.59	0.55	0.47	0.63	0.62	0.61	0.58	0.53

*ORR: odds ratio reduction; PF: prognostic factor.*

**Table 5:** Probability of difference between the estimated and true ORR≥0.10 in the unconditional setting scenario 3.

Difference from true ORR≥0.10		Unadjusted model					Adjusted model				
	Sample size	Prevalence of PF					Prevalence of PF				
		0.01	0.05	0.1	0.2	0.5	0.01	0.05	0.1	0.2	0.5
Over-	25	0.39	0.41	0.41	0.42	0.43	0.39	0.42	0.45	0.46	0.44
estimation	50	0.44	0.44	0.44	0.45	0.39	0.43	0.44	0.44	0.43	0.42
	125	0.38	0.39	0.39	0.38	0.36	0.39	0.40	0.39	0.39	0.37
	500	0.31	0.30	0.28	0.27	0.23	0.31	0.31	0.29	0.29	0.25
	1000	0.24	0.22	0.21	0.18	0.14	0.24	0.23	0.22	0.21	0.16
	2000	0.16	0.14	0.13	0.11	0.06	0.16	0.14	0.14	0.12	0.09
Under-	25	0.40	0.41	0.41	0.43	0.44	0.40	0.43	0.45	0.45	0.46
estimation	50	0.43	0.43	0.45	0.45	0.41	0.43	0.43	0.45	0.44	0.42
	125	0.41	0.39	0.39	0.39	0.37	0.42	0.41	0.40	0.39	0.38
	500	0.32	0.31	0.30	0.28	0.24	0.33	0.32	0.31	0.30	0.27
	1000	0.26	0.25	0.22	0.20	0.16	0.26	0.26	0.24	0.22	0.20
	2000	0.18	0.17	0.15	0.12	0.08	0.18	0.17	0.17	0.15	0.11
Overall	25	0.79	0.81	0.82	0.85	0.87	0.79	0.84	0.89	0.91	0.89
	50	0.86	0.88	0.89	0.89	0.80	0.86	0.88	0.88	0.87	0.84
	125	0.79	0.78	0.78	0.78	0.73	0.81	0.81	0.80	0.78	0.75
	500	0.63	0.61	0.58	0.54	0.47	0.64	0.63	0.60	0.59	0.52
	1000	0.49	0.47	0.43	0.38	0.30	0.50	0.49	0.47	0.43	0.36
	2000	0.34	0.30	0.27	0.23	0.15	0.34	0.32	0.30	0.27	0.20

*ORR: odds ratio reduction; PF: prognostic factor.*

In scenario 1, the distribution of the unadjusted ORR estimates was positively skewed, indicating a higher likelihood of underestimation than overestimation when PF was a strong predictor of the outcome and treatment was moderately efficacious. Adjusting for PF made the distribution of the ORR estimator symmetric around the true effect, i.e. random fluctuations were equally likely in either direction. When the influence of the PF was moderate or the actual treatment effect was zero, adjusting for PF did not improve accuracy or precision of the estimate.

#### Setup #2: the conditional setting

For all 8 scenarios in the conditional setting, the adjusted model produced roughly unbiased estimates of the treatment effect and maintained nominal coverage of the 95% CI. The unadjusted model overestimated treatment effects, and the model performance was influenced by multiple factors including the treatment effect, the effect and prevalence of the PF, and the sample size.

Figures S15 and S16 display the performances of the adjusted and unadjusted models in scenario 1 under the conditional setting with 125 and 2000 patients per arm. Ignoring the fact that 5% more patients had this PF in the control arm led to substantial overestimation of treatment effect. Bias was comparatively larger when PF was rare: when λ ranged between 0.05 and 0.2, bias of the unadjusted estimate of log OR, 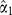, relative to 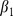 was between −0.18 and −0.09, with 125 per arm in scenario 1. Varying sample size led to little change in the magnitude of bias in scenario 1, though estimates were more variable with 125 or fewer patients per arm. Coverage of the unadjusted CI was greater than its nominal value with 125 or fewer per arm for most prevalence values between 0 and 1. The coverage reduced substantially as sample size went beyond 1000 per arm; when λ≤0.2 or≥0.8 coverage of the unadjusted CI dropped to 60%–90%. For a fixed sample size, the unadjusted estimate had slightly greater precision than the adjusted estimate; but the difference diminished as sample size increased.

With PF RR = 2 in scenario 2, bias of the unadjusted point estimator decreased with sample size and varied little with λ. The average biases of 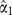 over the 11 prevalence values investigated were −0.014, −0.055 and −0.050 with 25, 50 and 125 patients per arm respectively and reduced to −0.015, −0.007 and −0.004 when the sample size reached 500, 1000 and 2000 per arm. The corresponding biases of the adjusted log OR estimator, 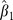, were 0.024, −0.024, −0.012, −0.005, −0.002 and −0.001. Both models achieved similar coverage when sample sizes were greater than or equal to 50 per arm, and demonstrated comparable precision.

The unadjusted model had slightly greater power though this advantage decreased as sample size increased. When the treatment had no effect, performance of the adjusted and unadjusted models in scenarios 3 and 4 was similar to that in scenario 2. Omitting a stronger PF in analysis again led to a greater bias for a fixed sample size and bias again shrank as trial size enlarged. Findings similar to scenarios 1–4 were demonstrated when the risk of outcome events in the control group reduced from 0.1 to 0.05 (scenarios 5–8). A low event rate in each trial resulted in reduced precision and statistical power. Bias of the unadjusted log OR estimates decreased with sample size and was generally smaller than the counterpart in the previous scenarios.

## Discussion

Our simulation results demonstrate that small sample size is associated with a high risk of imbalance in PFs in individual simple RCTs. The probabilities of an absolute imbalance ≥5% in a binary PF of prevalence 0.5 is 0.42, 0.62 and 0.67 with 125, 50 and 25 patients per arm. The probability of absolute imbalance decreases as sample size increases or prevalence of PF approaches 0 or 1.

Failing to adjust for a largely balanced strong PF (RR = 5) in a logistic regression model leads to bias toward no treatment effect when the actual size of treatment effect is moderate (RR = 0.75); this bias varies little with sample size greater than 50 patients per arm. Adjusting for such a PF reduces precision of the effect estimate but increases statistical power. The gain in power is comparatively larger when sample size is between 500 and 1000 per arm and prevalence is within 0.2–0.6, relative to other cases. When the PF is less powerful and a treatment difference exists, improvement in accuracy and efficiency associated with the adjustment for a largely balanced PF is less noticeable. When the treatment effect is zero, such covariate adjustment leads to minimal loss of precision. Overall the simulation results based on a single binary baseline PF suggest it is critical to adjust for important PFs in trials evaluating a binary outcome. If ignored, substantial bias due to confounding or non-collapsibility can emerge; bias would be more marked when PF has high predictive value and sample size is small to moderate.

It is challenging to establish a single rule for sample size requirement focused on the probability and impact of prognostic imbalance. Multiple factors influence the requirement.

Firstly, sample size should be sufficiently large that the probability of imbalance is restricted to a reasonably low value. The adequate sample size varies with the choice of imbalance measure, the size of imbalance that is deemed important, and the prevalence of the PF. For example, Figure 1 suggests that if an absolute measure of imbalance ≥0.05 is deemed important, 1000 patients per arm is a reasonable size.

Secondly, sample size should be sufficient to produce a reliable estimate of treatment effect. Although it has less impact on the magnitude of bias around the mean effect estimate in the unconditional setting, sample size does affect precision. While adjusting for PF removes systematic bias, estimates from an individual trial may still deviate from the true effect in either direction due to random sampling variation. Tables 2 and 4 suggest that probabilities of having an absolute deviation > = 0.05 (in either direction) from the true ORR are 0.87–0.93 and 0.52–0.62 for trials recruiting 125 and 2000 patients per arm, respectively. If trialists are willing to tolerate a slightly bigger deviation from the true ORR, for instance, no more than 0.1, the above probabilities decrease to 0.75–0.81 (125/arm) and 0.20–0.32 (2000/arm) for both models, and 2000 patients per arm then seems to be a reasonable sample size (Tables 3 and 5). As PF becomes less prevalent, larger trial sizes are required for purposes of precision. When randomization partially or completely fails, no statistical adjustment or increase in sample size can fully correct the resulting bias.

The current investigation on the likelihood of prognostic imbalance and its implications for sample size requirements is consistent with previous findings. A minimum of 100 patients per arm has been suggested to control the chance of imbalance of 20% or more in a single PF [31], and 1350 per arm may be needed to minimize the chance of a 5% imbalance [3]. Although Cui et al calculated the probabilities of a 20% imbalance in at least one out of *k* independent PFs (*k* = 2, 3, and 4) [31], situations involving multiple correlated PFs are worth further investigation.

Gail first demonstrated that omitting balanced baseline covariates in logistic regression asymptotically (i.e. for very large sample sizes) results in downward bias on the subject-specific treatment-outcome association [14]. This is also referred to as the non-collapsibility problem [16], because the odds ratio as the measure of association between the treatment and the binary outcome within each category of the baseline covariates (i.e. conditional or subject-specific association) is different from the association across all categories of the covariates (i.e. the marginal or average association).

In their simulation study [32], Negassa and Hanley showed that omitting an important balanced continuous or binary covariate in logistic regression model lowers both the coverage probability (that is, the proportion of the time that the CI contains the true value of interest in a set of hypothetical repetition of data collection and analysis procedure [33]) and study power in binary trials with moderate sample sizes (n = 500 and 1000). These findings are complemented by a simulation study that explored the effect of imbalance in two continuous baseline covariates on power in a logistic regression framework when both variables were adjusted for in analyzing small trials (n = 50, 100 and 300) [12]. Others quantified the increase in statistical power resulting from covariate adjustment as a decrease in the sample size required in comparison to the unadjusted model [11].

It was not clear in the literature, however, how the interplay of chance imbalance, the risk of outcome and the prevalence of a binary PF affects treatment effect estimation in trials with a binary outcome. Our simulation study provided information on what constitute an adequate sample size to control against potential impact of prognostic imbalance. Our results based on trials subject to chance imbalance across six sample sizes in the unconditional setting are consistent with the previous findings.

When one is confident that all important PFs are distributed similarly between treatment groups in a binary trial, it is sensible to decide if the goal of a trial evaluating a binary outcome is to assess the marginal effect of treatment over patients with heterogeneous baseline prognosis, or to obtain a more individualized treatment effect estimate that is specific to a prognosis. These objectives can be achieved by using the unadjusted and adjusted logistic regression analyses. With a binary outcome, the two models produce mathematically different results in the presence of a non-zero treatment effect. Mismatch of the study objective, the statistical method, and interpretation of results can result in misleading messages. Due to the uncertainty around the existence or magnitude of the treatment effect and possibly different criteria to assess prognostic imbalance, we recommend reporting both the adjusted and unadjusted results in the manuscript.

The CPMP guideline recommends that including important PFs in the primary analysis can be justified only if their associations with the primary outcome are expected to be strong, based on previous evidence, and are specified a priori [18]. What constitutes adequate justification may be a matter of judgment. Our results demonstrate the value of adjustment, and suggest the merits of avoiding excessively stringent criteria when deciding whether prior evidence of prognostic power is adequate.

Our study has several limitations. First, we included only one binary baseline PF to illustrate the probability and impact of prognostic imbalance in RCTs evaluating a binary outcome. For continuous PFs, Ciolino and colleagues proposed a rank-sum ratio to measure the level of imbalance in addition to the commonly used mean values [12]. When multiple PFs are present at baseline, balancing distribution of the individual PFs and the overall prognosis needs to be assessed. Although the single binary PF considered in the current study can be conceptualized as a measure of the overall prognosis of a patient based on multiple PFs, for instance, in a propensity score framework [34], further investigation on the distribution and impact of multiple correlated PFs on effect estimation in RCTs is warranted.

Second, although our investigation was focused on prognostic balancing in individual RCTs, systematic reviews and meta-analyses face the same methodological challenges. The cumulative number of patients from individual RCTs and the between-study variation need to be considered to assess the impact of imbalance on obtaining an aggregated estimate of treatment effects. Future work is needed in these directions.

Our study provides useful new insights. The results can not only help to design clinical trials, but can also inform quality assessment of a body of evidence from RCTs. Our simulation findings provide insights on prognostic imbalance which pertains to both risk of bias and imprecision [35]. The current study was not designed to propose a single threshold value of sample size that can be readily employed to rate the quality of evidence with respect to precision. Rather it lends itself to guide selection of such threshold values over various combinations of trial parameters, a subjective process likely influenced by the tolerance of risk.

In summary, prognostic imbalance does not on average jeopardize internal validity of findings from RCTs, but if neglected, may lead to chance confounding and biased estimate of treatment effect in a single RCT. To produce an accurate estimate of the treatment-outcome relationship conditional on patients' baseline prognosis, balanced or unbalanced PFs with high predictive value should be adjusted for in the analysis. Covariate adjustment slightly reduces precision, but improves study efficiency, when PFs are largely balanced. Once chance imbalance in baseline prognosis is observed, covariate adjustment should be performed to remove chance confounding.

## Supporting Information

Figure S1 **Probability of imbalance using standardized measure (D_2_) with different trial sizes.** Lines correspond to Pr (D_2_≥d_1_), where d_1_ = 0.005 (hollow circle), 0.01 (triangle), 0.025 (cross), 0.05 (X), 0.10 (diamond), 0.15 (inverted triangle), and 0.20 (filled circle), from the top to the bottom, respectively. Top left: 25/arm, top right: 50/arm, middle left: 125/arm, middle right: 500/arm, bottom left: 1000/arm, bottom right: 2000/arm.
(TIF)

Figure S2 **Bias, simulation standard deviation (SD), coverage proportion and statistical power for the unadjusted and adjusted logOR, in scenario 1, the unconditional setting, with 25 patients per arm.** The unadjusted model is indicated by the dotted line with hollow circles, and the adjusted model is indicated by the solid line with filled circles.
(TIF)

Figure S3 **Bias, simulation standard deviation (SD), coverage proportion and statistical power for the unadjusted and adjusted logOR, in scenario 1, the unconditional setting, with 50 patients per arm.** The unadjusted model is indicated by the dotted line with hollow circles, and the adjusted model is indicated by the solid line with filled circles.
(TIF)

Figure S4 **Bias, simulation standard deviation (SD), coverage proportion and statistical power for the unadjusted and adjusted logOR, in scenario 1, the unconditional setting, with 500 patients per arm.** The unadjusted model is indicated by the dotted line with hollow circles, and the adjusted model is indicated by the solid line with filled circles.
(TIF)

Figure S5 **Bias, simulation standard deviation (SD), coverage proportion and statistical power for the unadjusted and adjusted logOR, in scenario 1, the unconditional setting, with 1000 patients per arm.** The unadjusted model is indicated by the dotted line with hollow circles, and the adjusted model is indicated by the solid line with filled circles.
(TIF)

Figure S6 **Bias, simulation standard deviation (SD), coverage proportion and statistical power for the unadjusted and adjusted logOR, in scenario 2, the unconditional setting, with 25 patients per arm.** The unadjusted model is indicated by the dotted line with hollow circles, and the adjusted model is indicated by solid line with filled circles.
(TIF)

Figure S7 **Bias, simulation standard deviation (SD), coverage proportion and statistical power for the unadjusted and adjusted logOR, in scenario 2, the unconditional setting, with 50 patients per arm.** The unadjusted model is indicated by the dotted line with hollow circles, and the adjusted model is indicated by the solid line with filled circles.
(TIF)

Figure S8 **Bias, simulation standard deviation (SD), coverage proportion and statistical power for the unadjusted and adjusted logOR, in scenario 2, the unconditional setting, with 125 patients per arm.** The unadjusted model is indicated by the dotted line with hollow circles, and the adjusted model is indicated by solid line with filled circles.
(TIF)

Figure S9 **Bias, simulation standard deviation (SD), coverage proportion and statistical power for the unadjusted and adjusted logOR, in scenario 2, the unconditional setting, with 2000 patients per arm.** The unadjusted model is indicated by the dotted line with hollow circles, and the adjusted model is indicated by the solid line with filled circles.
(TIF)

Figure S10 **Bias, simulation standard deviation (SD), coverage proportion and statistical power for the unadjusted and adjusted logOR, in scenario 3, the unconditional setting, with 125 patients per arm.** The unadjusted model is indicated by the dotted line with hollow circles, and the adjusted model is indicated by the solid line with filled circles.
(TIF)

Figure S11 **Probability of difference between the estimated and true ORR (underestimation) in scenario 1, the unconditional setting, with 125 patients per arm.** Within each graph, lines correspond to Pr (D_ORR_≤−d_2_), where d_2_ = 0 (solid circle), 0.05 (bullet), 0.10 (little circle), 0.15 (square), 0.2 (diamond) and 0.25 (triangle), from top to bottom, respectively.
(TIF)

Figure S12 **Probability of difference between the estimated and true ORR (overestimation) in scenario 1, the unconditional setting, with 125 patients per arm.** Within each graph, lines correspond to Pr (D_ORR_≥d_2_), where d_2_ = 0 (solid circle), 0.05 (bullet), 0.10 (little circle), 0.15 (square), 0.2 (diamond) and 0.25 (triangle), from top to bottom, respectively.
(TIF)

Figure S13 **Probability of difference between the estimated and true ORR (underestimation) in scenario 1, the unconditional setting, with 2000 patients per arm.** Within each graph, lines correspond to Pr (D_ORR_≤−d_2_), where d_2_ = 0 (solid circle), 0.05 (bullet), 0.10 (little circle), 0.15 (square), 0.2 (diamond) and 0.25 (triangle), from top to bottom, respectively.
(TIF)

Figure S14 **Probability of difference between the estimated and true ORR (overestimation) in scenario 1, the unconditional setting, with 2000 patients per arm.** Within each graph, lines correspond to Pr (D_ORR_≥d_2_), where d_2_ = 0 (solid circle), 0.05 (bullet), 0.10 (little circle), 0.15 (square), 0.2 (diamond) and 0.25 (triangle), from top to bottom, respectively.
(TIF)

Figure S15 **Bias, simulation standard deviation (SD), coverage proportion and statistical power for the unadjusted and adjusted logOR, in scenario 1, the conditional setting, with 125 patients per arm.** The unadjusted model is indicated by the dotted line with hollow circles, and the adjusted model is indicated by the solid line with filled circles.
(TIF)

Figure S16 **Bias, simulation standard deviation (SD), coverage proportion and statistical power for the unadjusted and adjusted logOR, in scenario 1, the conditional setting, with 2000 patients per arm.** The unadjusted model is indicated by the dotted line with hollow circles, and the adjusted model is indicated by the solid line with filled circles.
(TIF)
