# Supplementary figures and images for: Assessment and Implication of Prognostic Imbalance in Randomized Controlled Trials with a Binary Outcome – A Simulation Study
Source: PLoS One. 2012 May 22;7(5):e36677. doi: 10.1371/journal.pone.0036677 (PMC3358303; doi:10.1371/journal.pone.0036677)

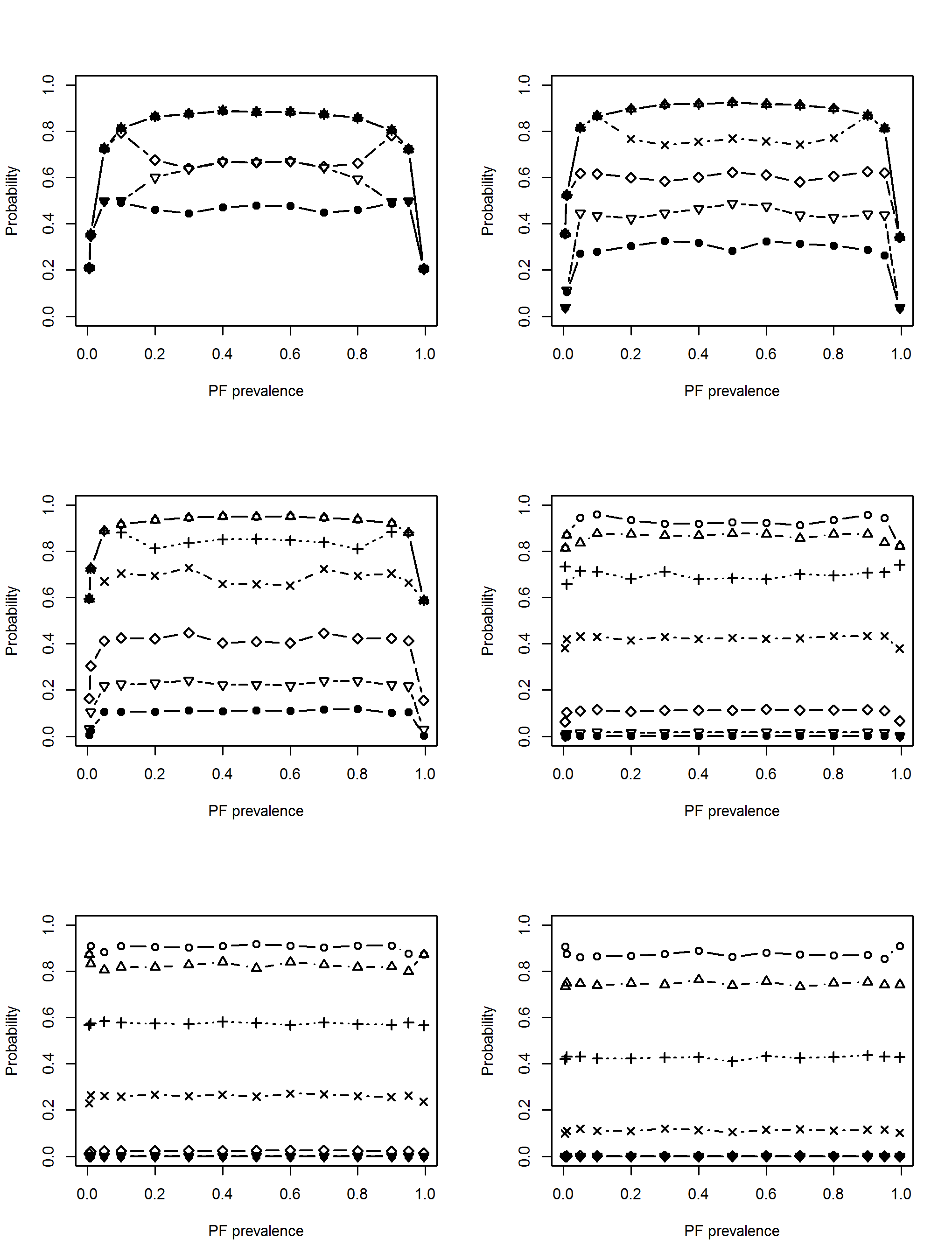

Supplement: Figure S1 — Probability of imbalance using standardized measure (D2) with different trial sizes. Lines correspond to Pr (D2≥d1), where d1 = 0.005 (hollow circle), 0.01 (triangle), 0.025 (cross), 0.05 (X), 0.10 (diamond), 0.15 (inverted triangle), and 0.20 (filled circle), from the top to the bottom, respectively. Top left: 25/arm, top right: 50/arm, middle left: 125/arm, middle right: 500/arm, bottom left: 1000/arm, bottom right: 2000/arm. (TIF) [file pone.0036677.s001.tif]

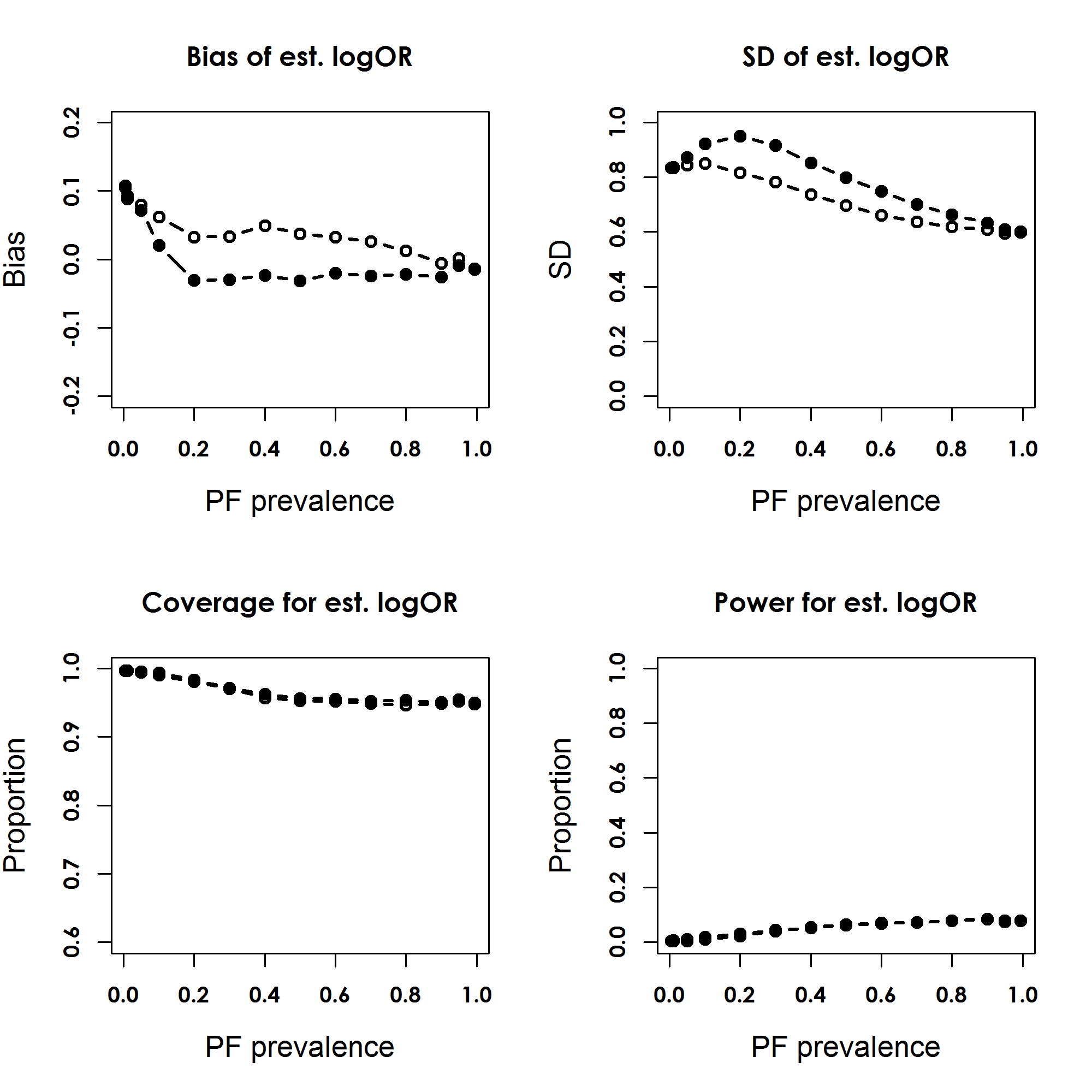

Supplement: Figure S2 — Bias, simulation standard deviation (SD), coverage proportion and statistical power for the unadjusted and adjusted logOR, in scenario 1, the unconditional setting, with 25 patients per arm. The unadjusted model is indicated by the dotted line with hollow circles, and the adjusted model is indicated by the solid line with filled circles. (TIF) [file pone.0036677.s002.tif]

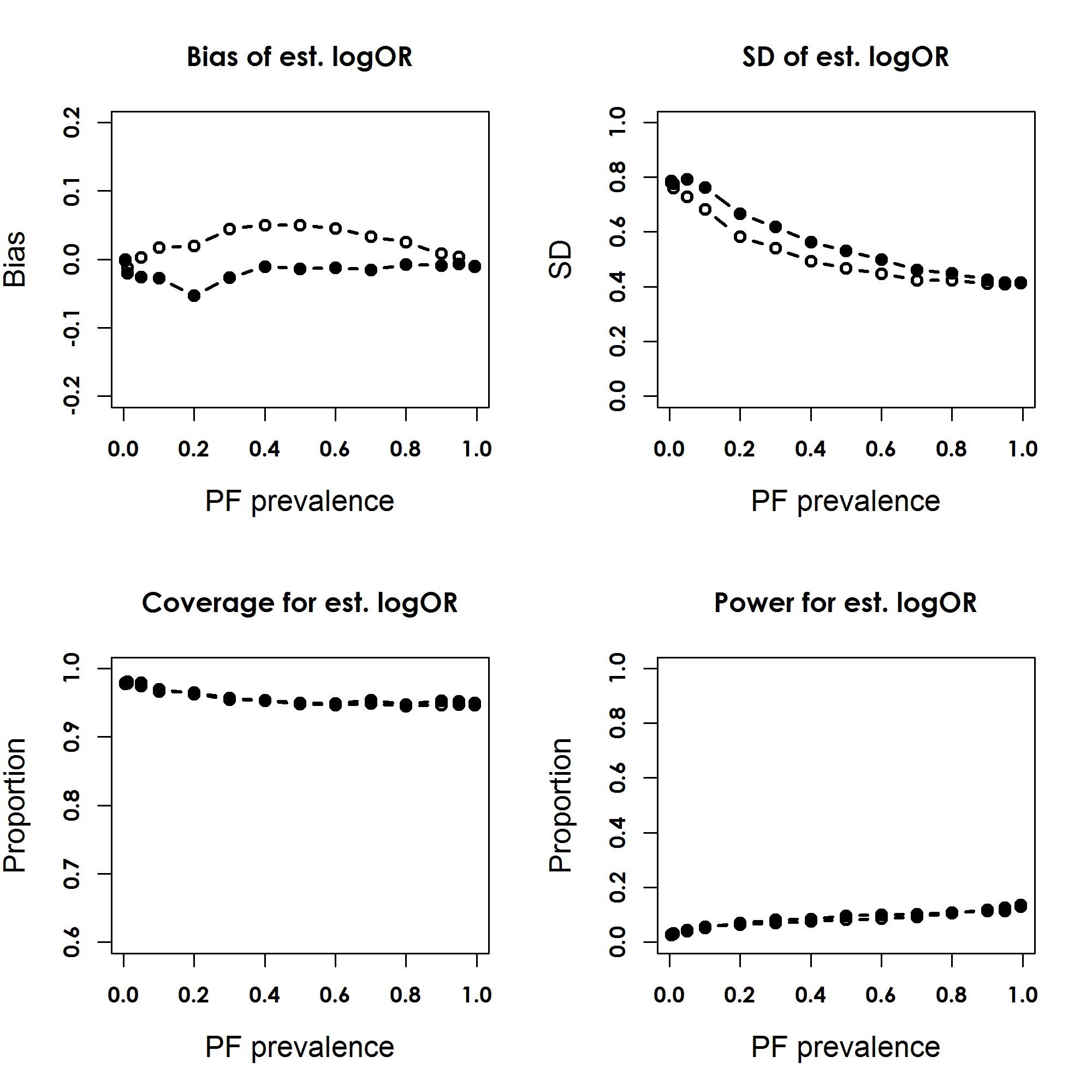

Supplement: Figure S3 — Bias, simulation standard deviation (SD), coverage proportion and statistical power for the unadjusted and adjusted logOR, in scenario 1, the unconditional setting, with 50 patients per arm. The unadjusted model is indicated by the dotted line with hollow circles, and the adjusted model is indicated by the solid line with filled circles. (TIF) [file pone.0036677.s003.tif]

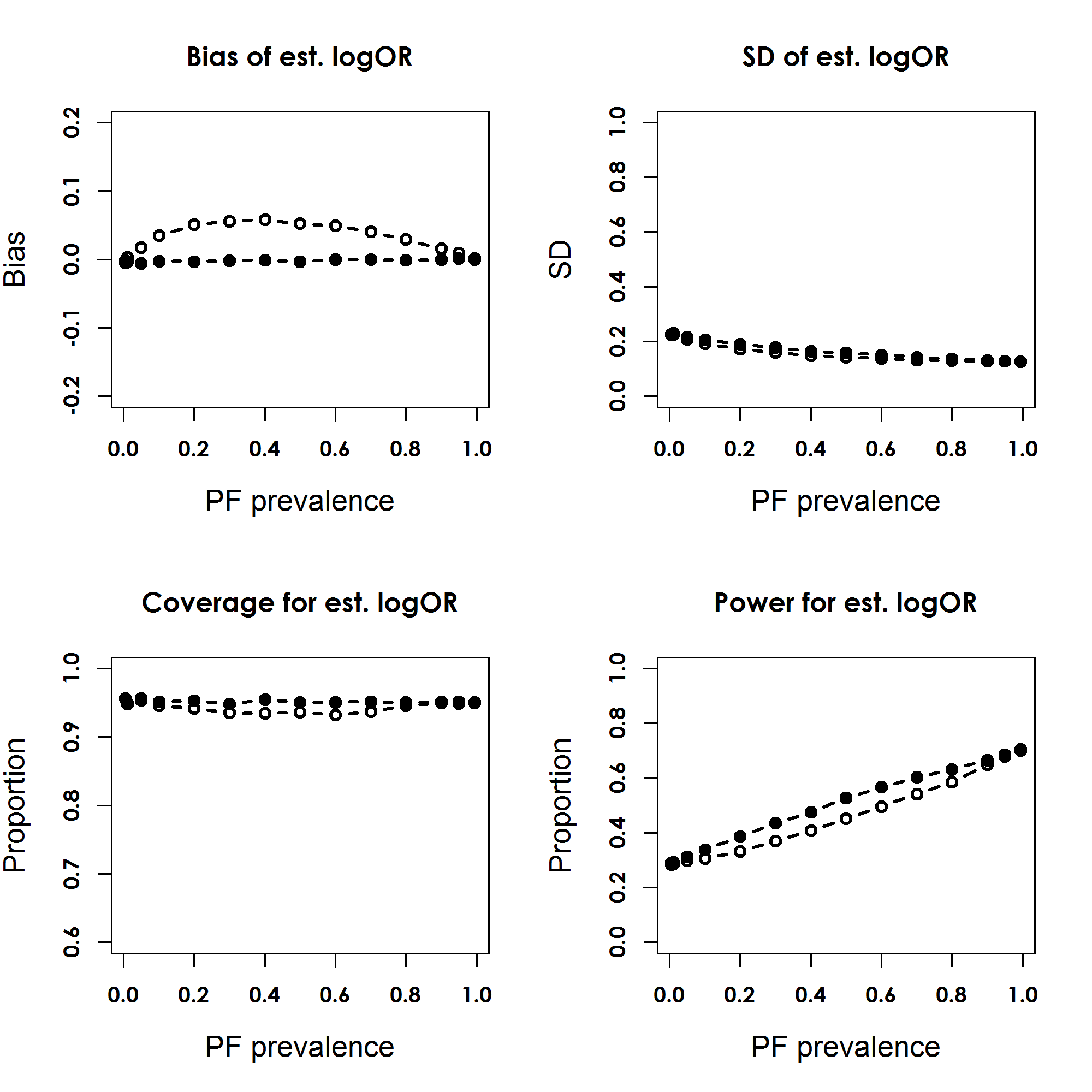

Supplement: Figure S4 — Bias, simulation standard deviation (SD), coverage proportion and statistical power for the unadjusted and adjusted logOR, in scenario 1, the unconditional setting, with 500 patients per arm. The unadjusted model is indicated by the dotted line with hollow circles, and the adjusted model is indicated by the solid line with filled circles. (TIF) [file pone.0036677.s004.tif]

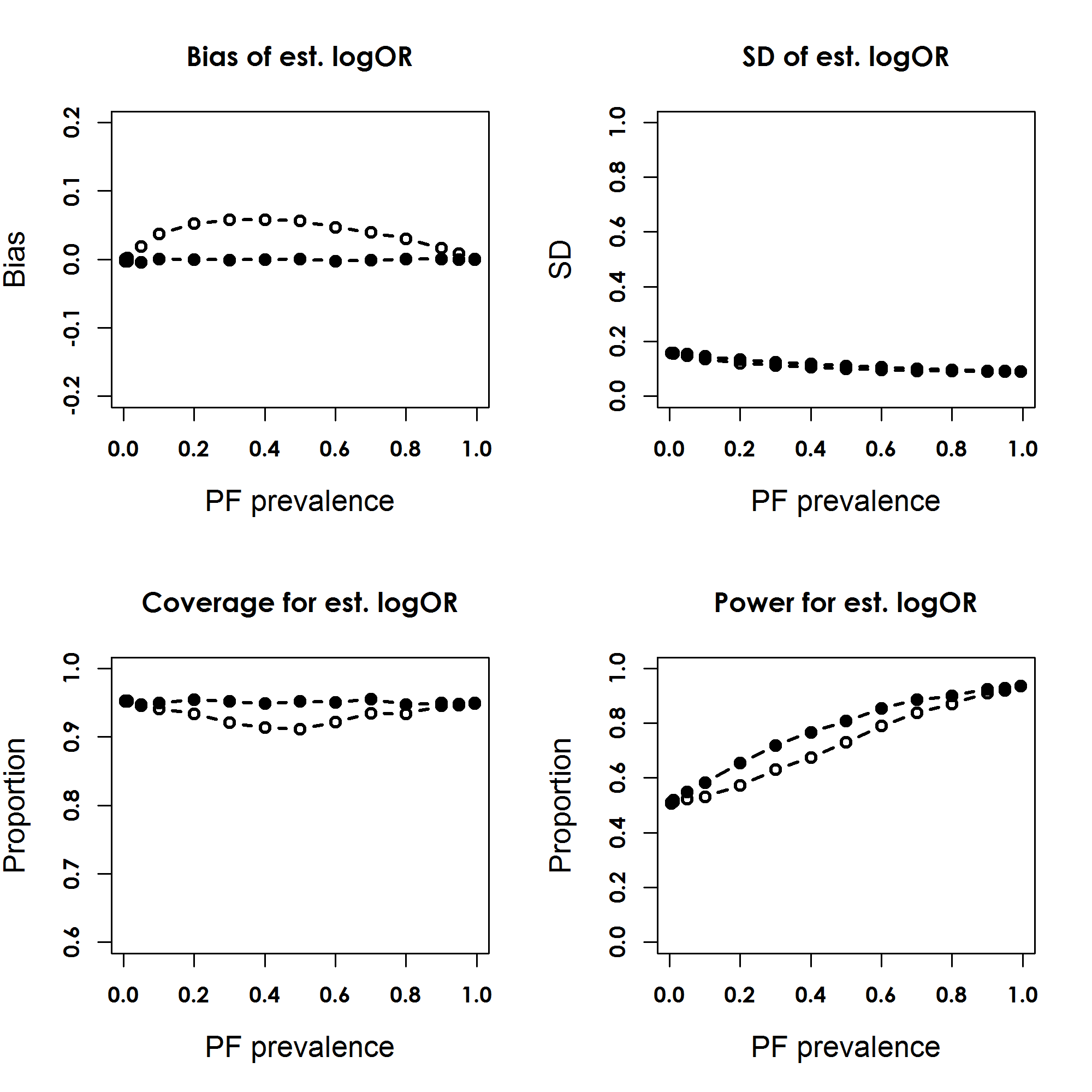

Supplement: Figure S5 — Bias, simulation standard deviation (SD), coverage proportion and statistical power for the unadjusted and adjusted logOR, in scenario 1, the unconditional setting, with 1000 patients per arm. The unadjusted model is indicated by the dotted line with hollow circles, and the adjusted model is indicated by the solid line with filled circles. (TIF) [file pone.0036677.s005.tif]

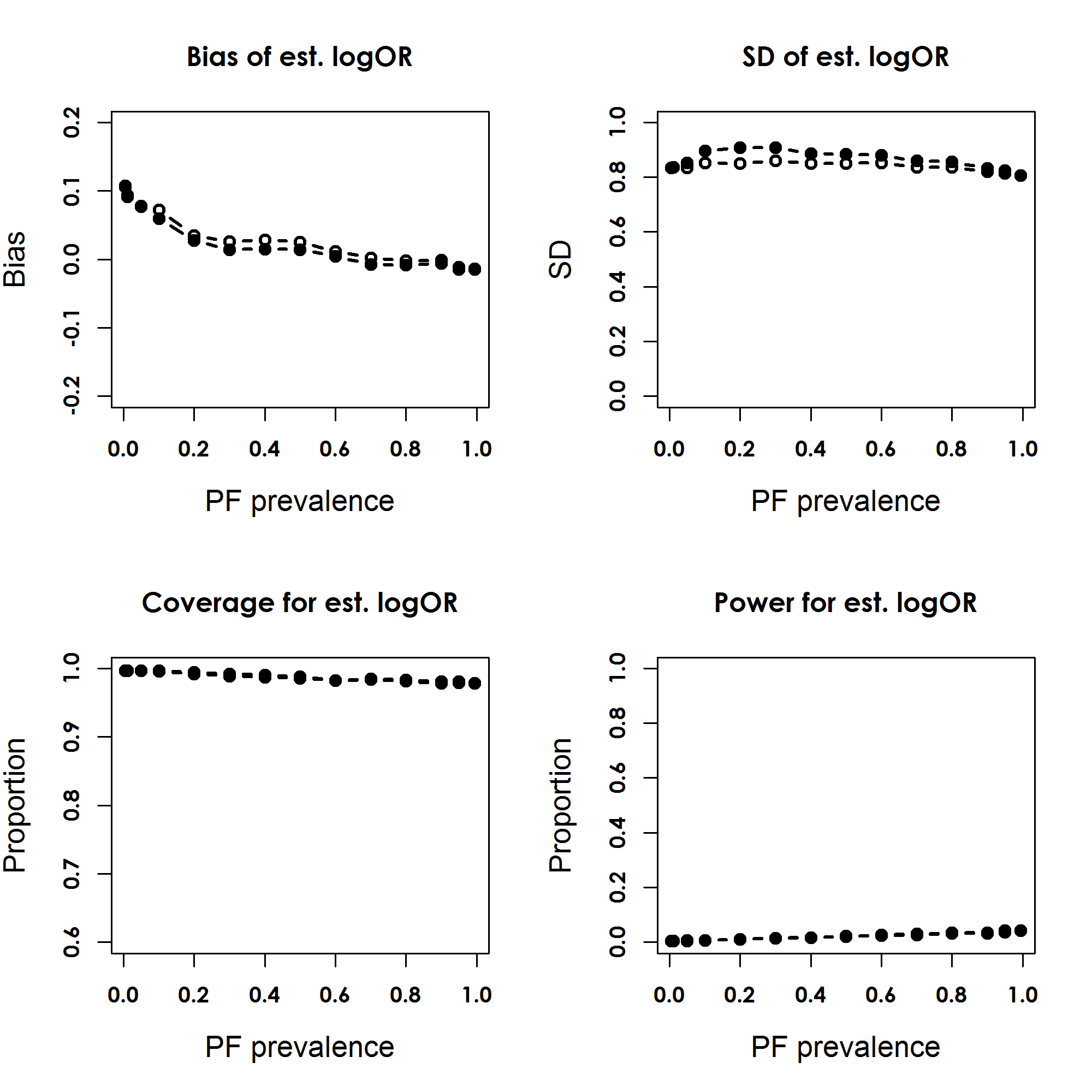

Supplement: Figure S6 — Bias, simulation standard deviation (SD), coverage proportion and statistical power for the unadjusted and adjusted logOR, in scenario 2, the unconditional setting, with 25 patients per arm. The unadjusted model is indicated by the dotted line with hollow circles, and the adjusted model is indicated by solid line with filled circles. (TIF) [file pone.0036677.s006.tif]

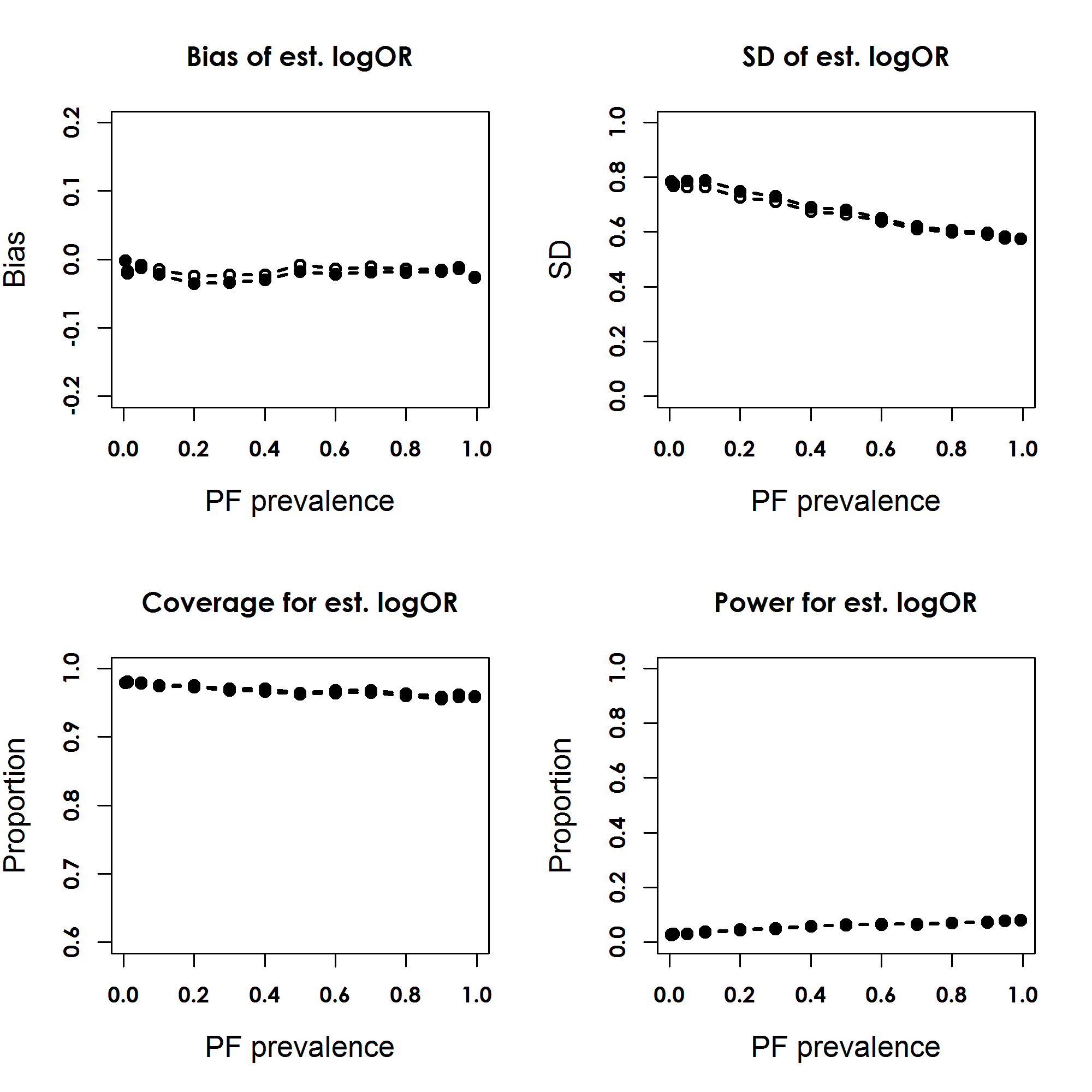

Supplement: Figure S7 — Bias, simulation standard deviation (SD), coverage proportion and statistical power for the unadjusted and adjusted logOR, in scenario 2, the unconditional setting, with 50 patients per arm. The unadjusted model is indicated by the dotted line with hollow circles, and the adjusted model is indicated by the solid line with filled circles. (TIF) [file pone.0036677.s007.tif]

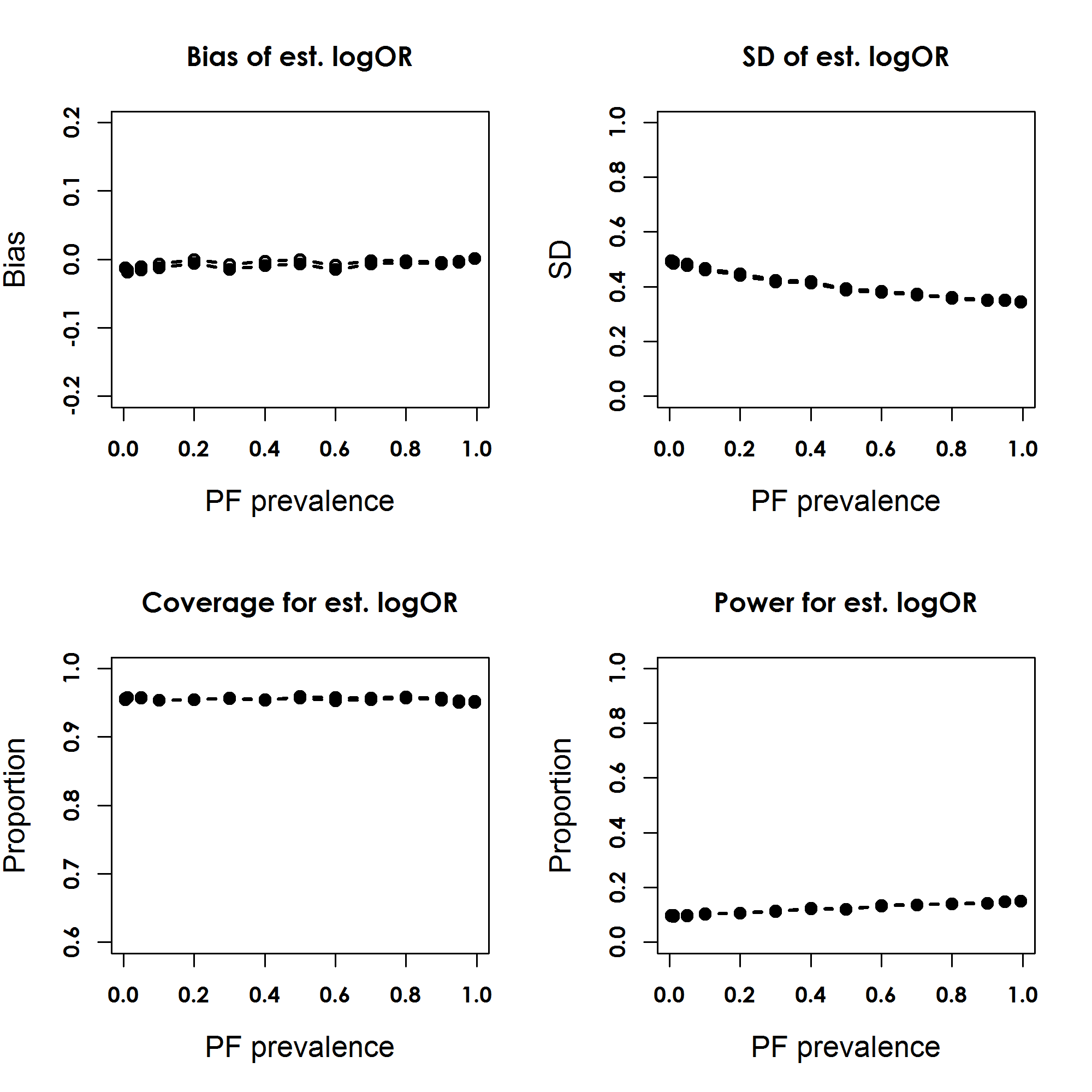

Supplement: Figure S8 — Bias, simulation standard deviation (SD), coverage proportion and statistical power for the unadjusted and adjusted logOR, in scenario 2, the unconditional setting, with 125 patients per arm. The unadjusted model is indicated by the dotted line with hollow circles, and the adjusted model is indicated by solid line with filled circles. (TIF) [file pone.0036677.s008.tif]

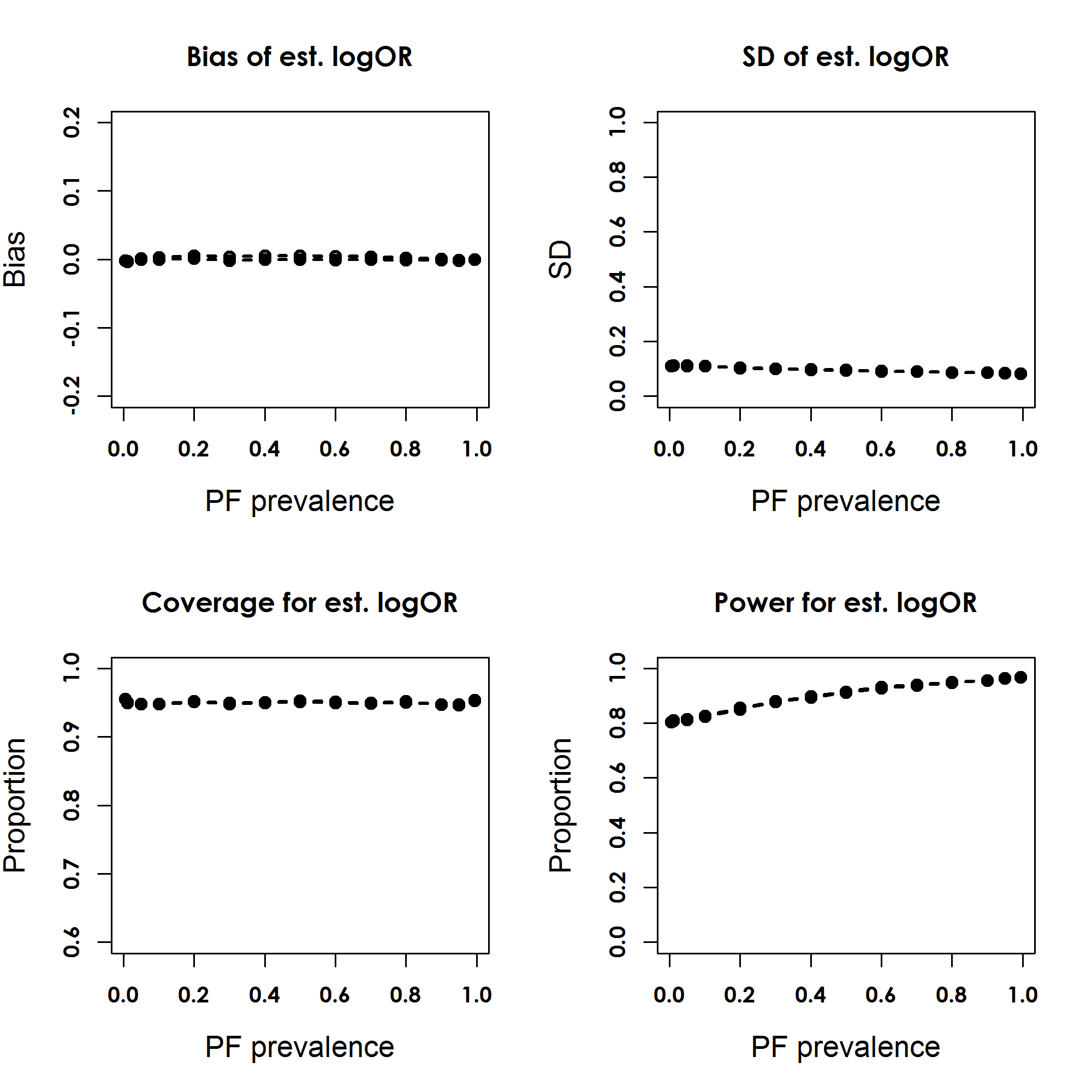

Supplement: Figure S9 — Bias, simulation standard deviation (SD), coverage proportion and statistical power for the unadjusted and adjusted logOR, in scenario 2, the unconditional setting, with 2000 patients per arm. The unadjusted model is indicated by the dotted line with hollow circles, and the adjusted model is indicated by the solid line with filled circles. (TIF) [file pone.0036677.s009.tif]

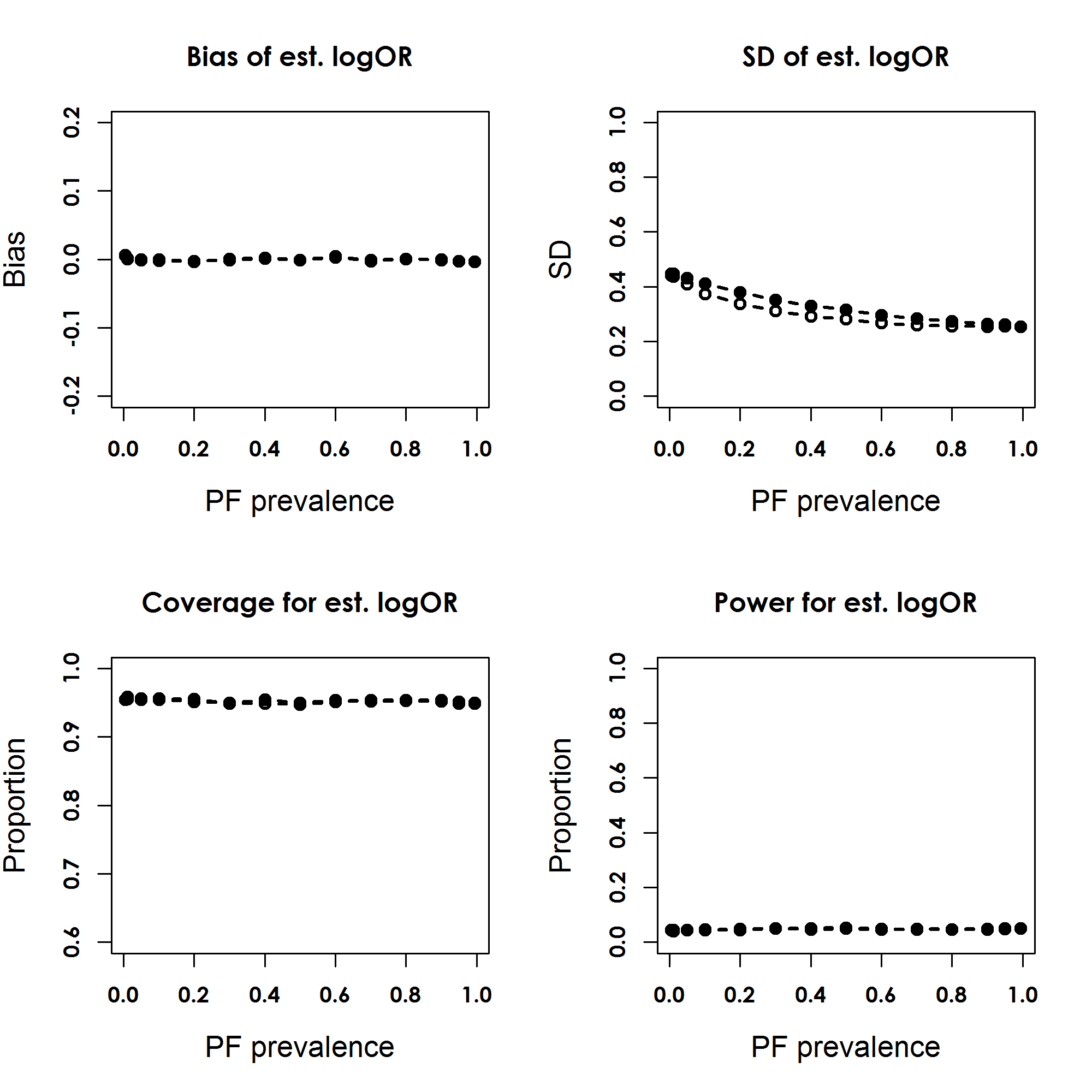

Supplement: Figure S10 — Bias, simulation standard deviation (SD), coverage proportion and statistical power for the unadjusted and adjusted logOR, in scenario 3, the unconditional setting, with 125 patients per arm. The unadjusted model is indicated by the dotted line with hollow circles, and the adjusted model is indicated by the solid line with filled circles. (TIF) [file pone.0036677.s010.tif]

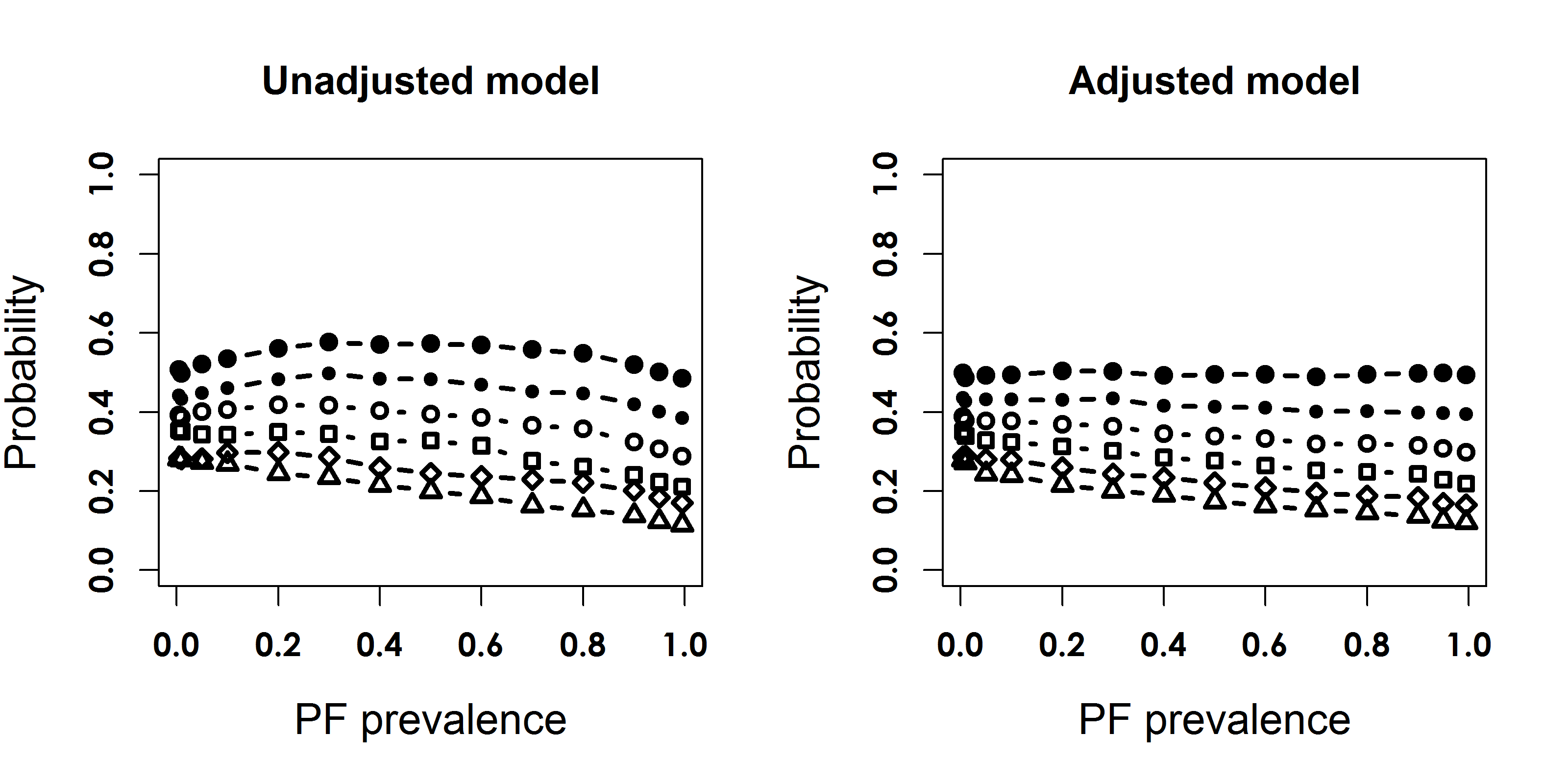

Supplement: Figure S11 — Probability of difference between the estimated and true ORR (underestimation) in scenario 1, the unconditional setting, with 125 patients per arm. Within each graph, lines correspond to Pr (DORR≤−d2), where d2 = 0 (solid circle), 0.05 (bullet), 0.10 (little circle), 0.15 (square), 0.2 (diamond) and 0.25 (triangle), from top to bottom, respectively. (TIF) [file pone.0036677.s011.tif]

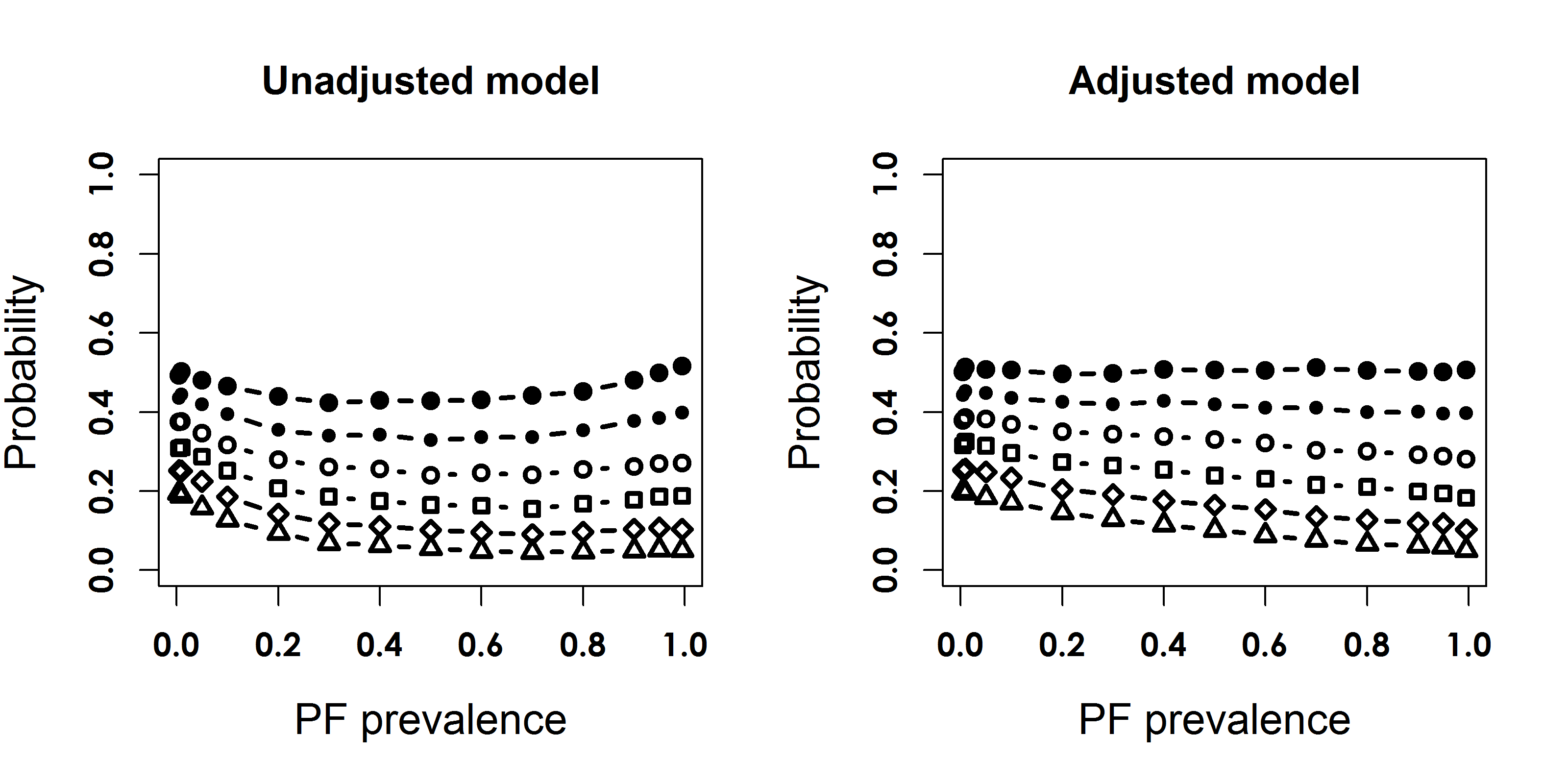

Supplement: Figure S12 — Probability of difference between the estimated and true ORR (overestimation) in scenario 1, the unconditional setting, with 125 patients per arm. Within each graph, lines correspond to Pr (DORR≥d2), where d2 = 0 (solid circle), 0.05 (bullet), 0.10 (little circle), 0.15 (square), 0.2 (diamond) and 0.25 (triangle), from top to bottom, respectively. (TIF) [file pone.0036677.s012.tif]

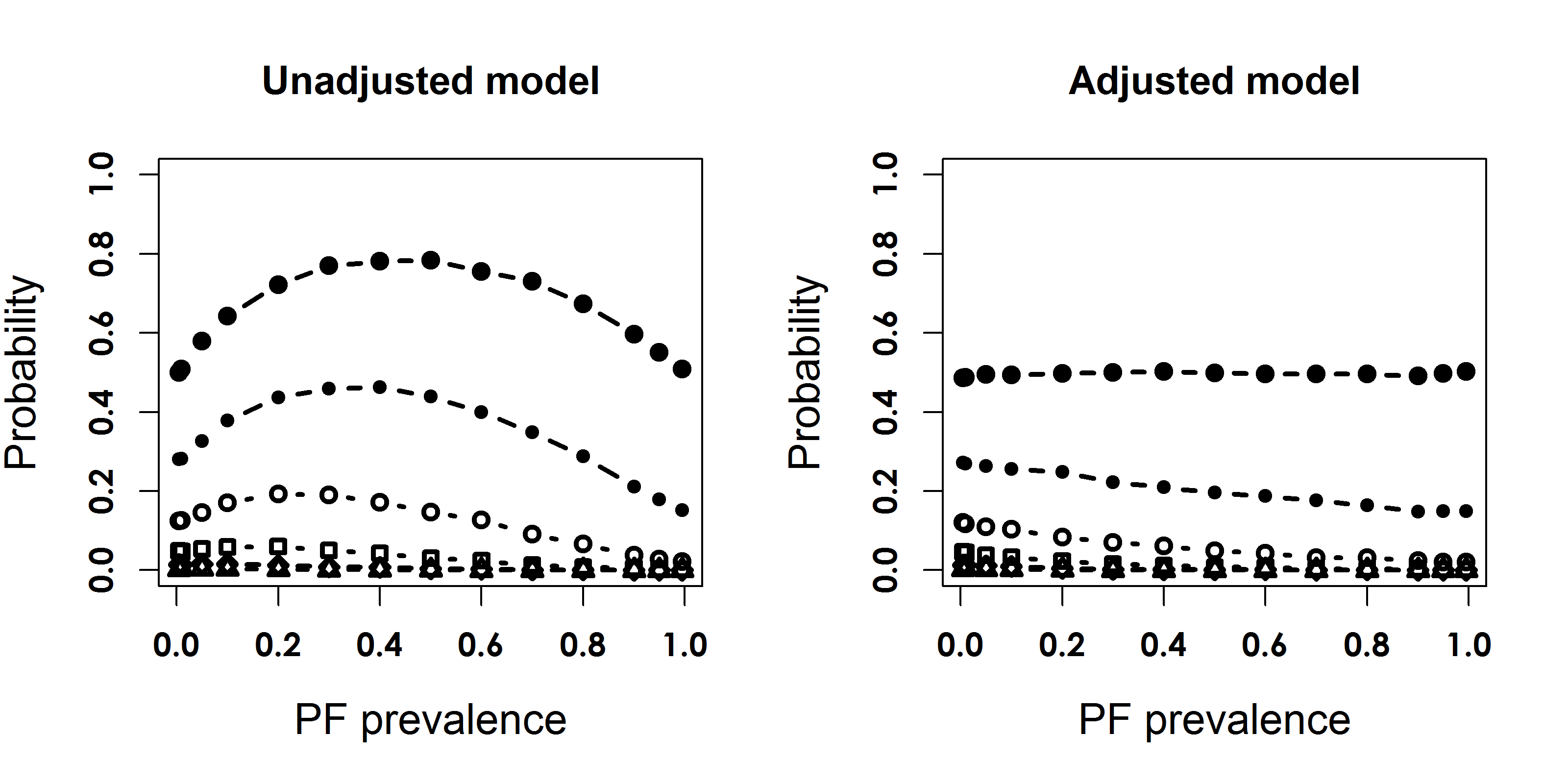

Supplement: Figure S13 — Probability of difference between the estimated and true ORR (underestimation) in scenario 1, the unconditional setting, with 2000 patients per arm. Within each graph, lines correspond to Pr (DORR≤−d2), where d2 = 0 (solid circle), 0.05 (bullet), 0.10 (little circle), 0.15 (square), 0.2 (diamond) and 0.25 (triangle), from top to bottom, respectively. (TIF) [file pone.0036677.s013.tif]

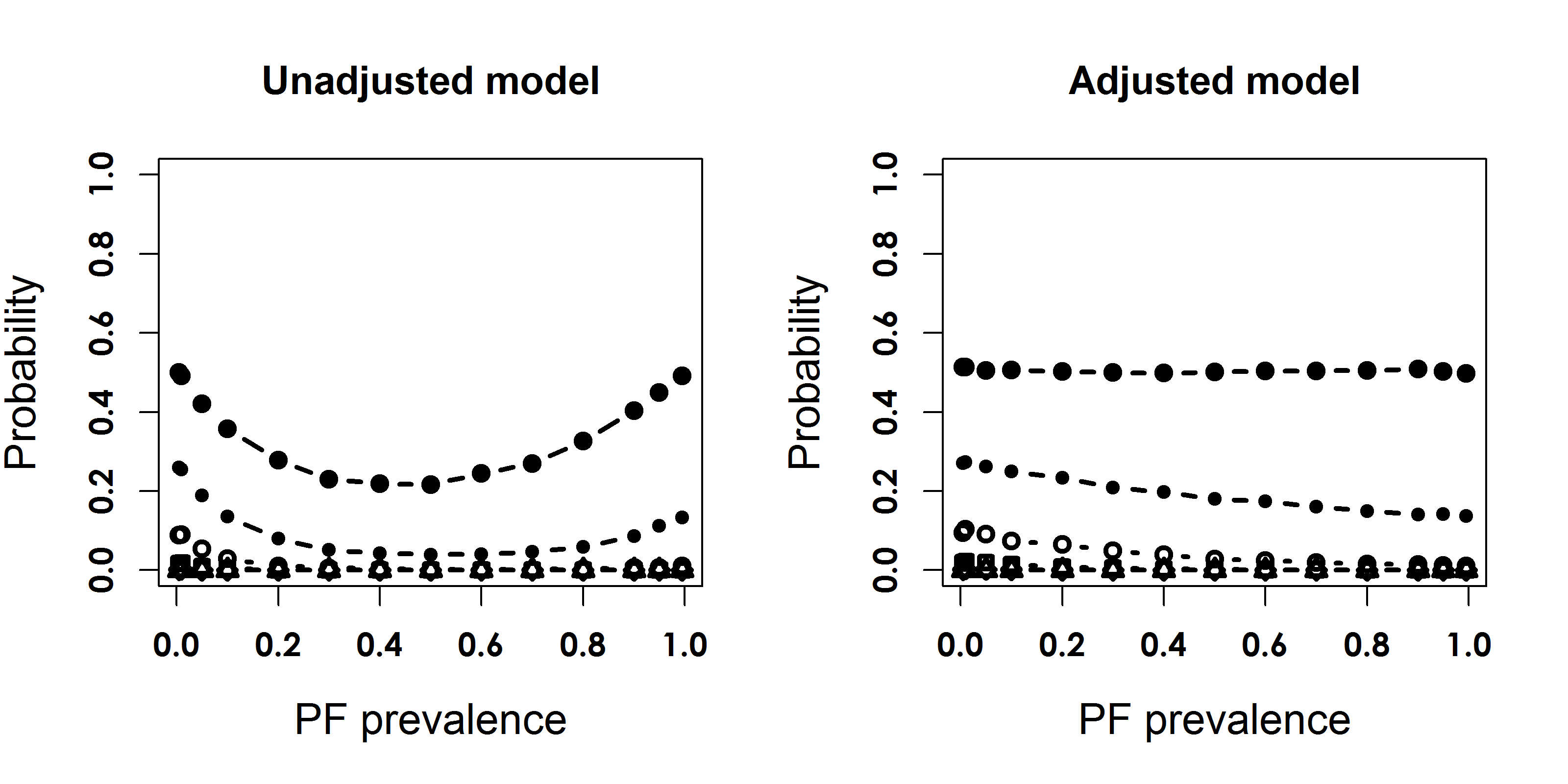

Supplement: Figure S14 — Probability of difference between the estimated and true ORR (overestimation) in scenario 1, the unconditional setting, with 2000 patients per arm. Within each graph, lines correspond to Pr (DORR≥d2), where d2 = 0 (solid circle), 0.05 (bullet), 0.10 (little circle), 0.15 (square), 0.2 (diamond) and 0.25 (triangle), from top to bottom, respectively. (TIF) [file pone.0036677.s014.tif]

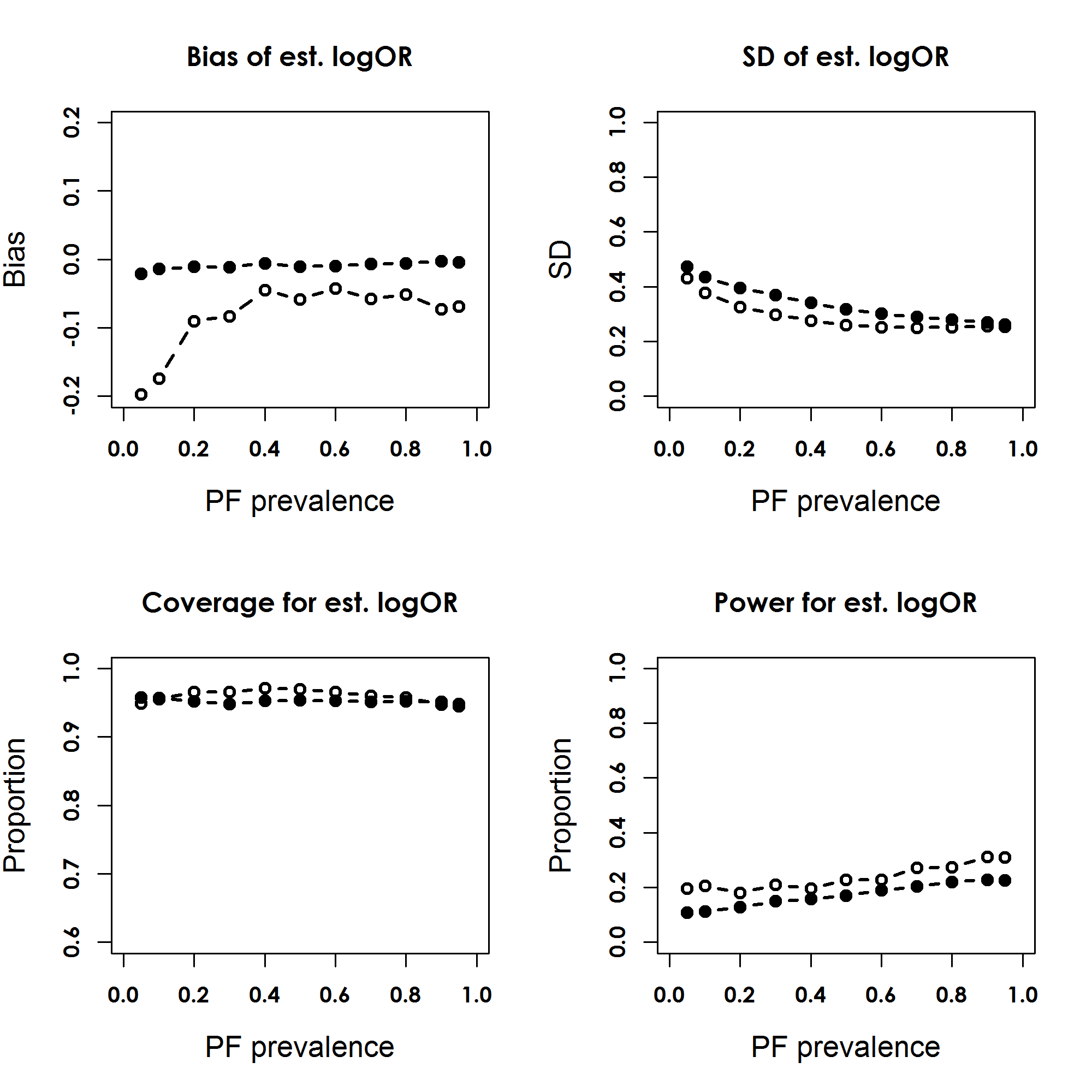

Supplement: Figure S15 — Bias, simulation standard deviation (SD), coverage proportion and statistical power for the unadjusted and adjusted logOR, in scenario 1, the conditional setting, with 125 patients per arm. The unadjusted model is indicated by the dotted line with hollow circles, and the adjusted model is indicated by the solid line with filled circles. (TIF) [file pone.0036677.s015.tif]

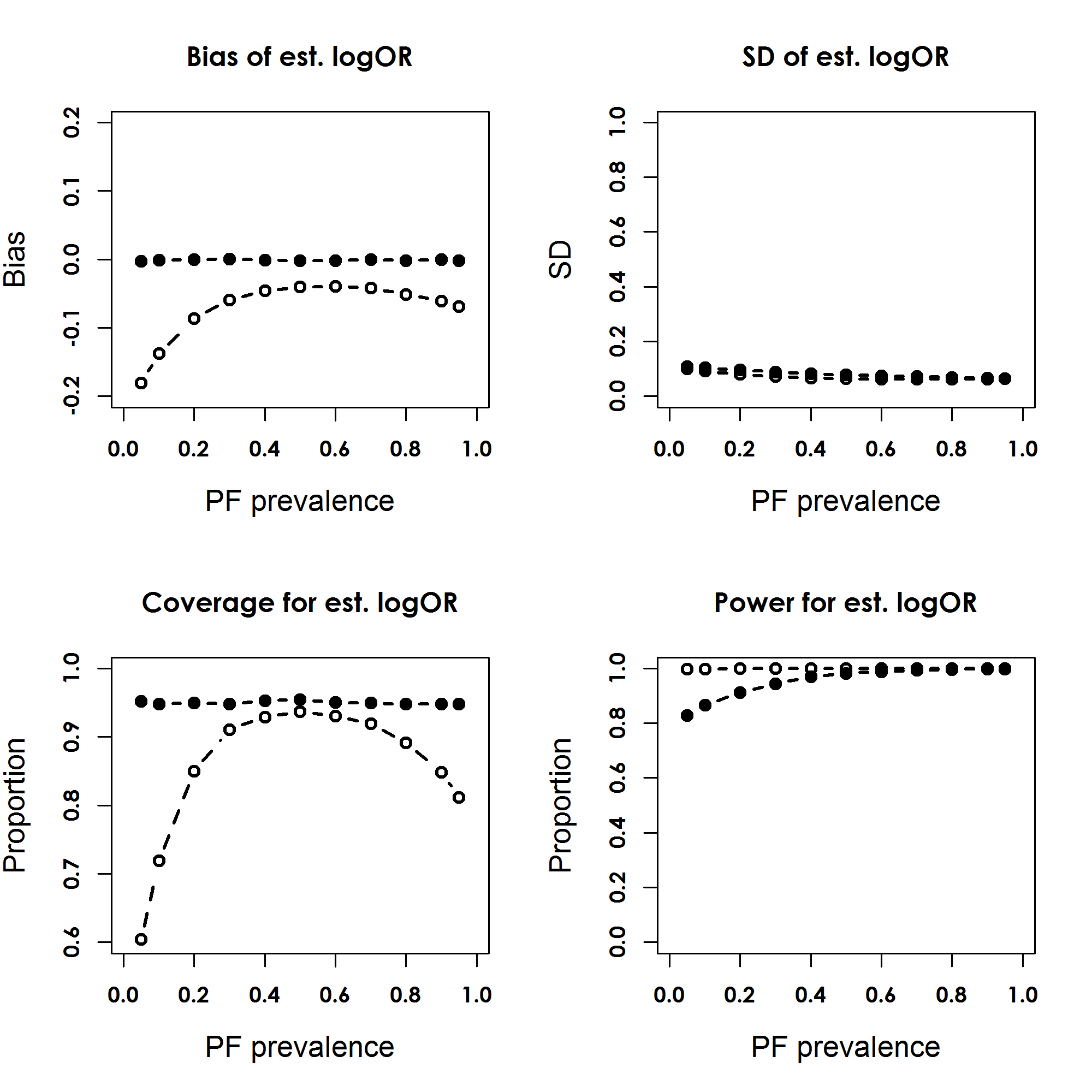

Supplement: Figure S16 — Bias, simulation standard deviation (SD), coverage proportion and statistical power for the unadjusted and adjusted logOR, in scenario 1, the conditional setting, with 2000 patients per arm. The unadjusted model is indicated by the dotted line with hollow circles, and the adjusted model is indicated by the solid line with filled circles. (TIF) [file pone.0036677.s016.tif]
